# The Structure of the Toronto Alexithymia Scale (TAS-20): A Meta-Analytic Confirmatory Factor Analysis

**DOI:** 10.1177/10731911211033894

**Published:** 2021-07-26

**Authors:** Ulrich Schroeders, Fiona Kubera, Timo Gnambs

**Affiliations:** 1 University of Kassel, Kassel, Hesse, Germany; 2 Leibniz Institute for Educational Trajectories, Bamberg, Bavaria, Germany

**Keywords:** Alexithymia, TAS-20, meta-analytic structural equation modeling, dimensionality, psychometrics

## Abstract

Alexithymia is defined as the inability of persons to describe their emotional states, to identify the feelings of others, and a utilitarian type of thinking. The most popular instrument to assess alexithymia is the Toronto Alexithymia Scale (TAS-20). Despite its widespread use, an ongoing controversy pertains to its internal structure. The TAS-20 was originally constructed to capture three different factors, but several studies suggested different factor solutions, including bifactor models and models with a method factor for the reversely keyed items. The present study examined the dimensionality of the TAS-20 using summary data of 88 samples from 62 studies (total *N* = 69,722) with meta-analytic structural equation modeling. We found support for the originally proposed three-dimensional solution, whereas more complex models produced inconsistent factor loadings. Because a major source of misfit stems from translated versions, the results are discussed with respect to generalizations across languages and cultural contexts.

*Alexithymia*—composed of the Greek *a* = lack, *lexis* = word, and *thymos* = mood or emotion—can be literally translated with the inability to read and express emotions. The term was coined by the psychotherapists Sifneos and Nemiah to summarize symptoms they noticed in patients with psychosomatic illnesses (Apfel & Sifneos, 1979; Nemiah & Sifneos, 1970; Sifneos, 1973). As suggested by the etymology, the authors were struck by the incapacity of many of their patients to express or describe their feelings or the emotional states of others. They also noticed that these patients exhibited a rather utilitarian type of thinking which links to another psychoanalytic concept, namely that of operatory thinking. “La pensée opératoire” (operatory thinking) was introduced by the French psychoanalysts Marty and M’Uzan (1963) to characterize a mundane, unimaginative, and utilitarian type of thought processing. Thus, the clinical picture of alexithymia combines two strands: the emotional deficits to describe, to recognize, and distinguish between feelings, and the cognitive inability to go beyond a utilitarian and pragmatic view. Alexithymia itself is not a psychiatric disorder that has been codified in international classification systems such as the *DSM-5* or the ICD-10. Nonetheless, there are many psychiatric disorders overlapping with alexithymia. While the prevalence of alexithymia in the general population is about 10% (Franz et al., 2008; Honkalampi et al., 2000; Mattila et al., 2008), the prevalence is significantly elevated for several psychiatric disorders highlighting the importance of the construct: about 60% in anorexia nervosa and bulimia nervosa (Cochrane et al., 1993; Corcos et al., 2000), 27% up to 50% in major depressive disorder (Leweke et al., 2012), 34% in panic disorder (Cox et al., 1995), 50% in substance abusers (Taylor et al., 1990). Moreover, alexithymia is also associated with some personality disorders. For example, Spitzer et al. (2005) characterized high-alexithymic patients as cold, hostile, and socially avoidant. Not only overlaps with psychiatric diseases were reported but also links with a wide set of physical illnesses ranging from inflammatory bowel disease (30%, Iglesias-Rey et al., 2012) to Parkinson’s disease (21%, Costa et al., 2010). However, the cited prevalence rates are point estimates of individual studies with the typically small and selective samples (e.g., geographical, age distribution, etc.).

The construct of alexithymia is more strongly tied to a specific measurement instrument than almost any other—the Toronto Alexithymia Scale (TAS-20, Bagby et al., 1994), even though there are other instruments that have a similar scope such as the Bermond–Vorst Alexithymia Questionnaire (BVAQ, Vorst & Bermond, 2001) or the Perth Alexithymia Questionnaire (PAQ, Preece, Becerra, Robinson, Dandy, & Allan, 2018). As one of the first self-report measures for alexithymia, the TAS-20 has clearly contributed to the popularization of the construct worldwide. However, it has also often been criticized for its internal structure (e.g., Haviland & Reise, 1996; Kooiman et al., 2002), its reliability (e.g., Gignac et al., 2007), and validity (e.g., Leising et al., 2009; Marchesi et al., 2014). Despite these substantive criticisms, the TAS-20 is a popular measure which is used in many clinical contexts. A large number of studies was devoted to the question of the psychometric quality and the dimensionality of the questionnaire, resulting in a vivid cycle of proof and counterproof of a specific factor solution (e.g., Bagby et al., 2007; Gignac et al., 2007; Kooiman et al., 2002; Meganck et al., 2008). The present study aims to summarize the available findings meta-analytically and shed more light on potential sources of model misfit. To do so, we take a closer look at the measurement invariance of the TAS-20 across different language versions and the clinical status of patients and nonpatients.

## The Toronto Alexithymia Scales

**Figure 1.**
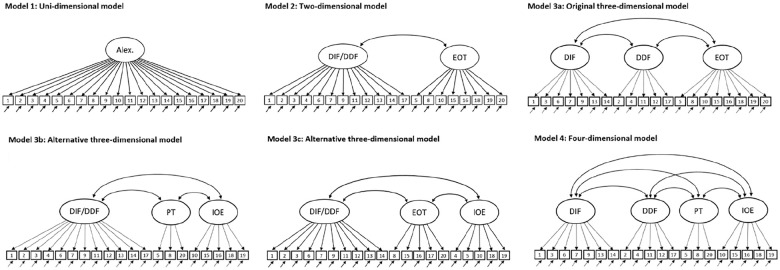
Competing measurement models for the TAS-20. *Note*. Please note that for Model 3c the factor labels were retained, although the items load on other factors compared with the standard solution. Alex. = Alexithymia; DIF = difficulty identifying feelings; DDF = difficulty describing feelings; EOT = external-oriented thinking; PT = pragmatic thinking; IOE = lack of (subjective significance or) importance of emotions.

The initial version of the TAS consists of 26 items with an equal number of negatively and positively keyed items to control for acquiescence (Bagby et al., 1986; Taylor et al., 1985). Factor analysis yielded a four-factor solution for the TAS-26 describing (a) the ability to identify and distinguish between feelings and bodily sensations, (b) the ability to describe feelings, (c) daydreaming, and (d) external-oriented thinking (EOT). Subsequently, a shortened version was created, the TAS-R (Taylor et al., 1992) with 23 items and an intended two-dimensional structure. Almost at the same time, the authors proposed a slightly reduced version, the TAS-20 (Bagby et al., 1994), which had better psychometric properties than the other versions of the TAS series. By now, 25 years after its initial presentation, the TAS-20 is the most popular measure (Bagby et al., 2020). The scale was designed to assess the three factors: (a) difficulty identifying feelings (DIF), (b) difficulty describing feelings (DDF), and (c) EOT. However, in the long history of its application, alternative factor structures have been proposed for the TAS-20 that we will shortly review in the following. The models are sorted from simple to complex (see Figure 1).

The unidimensional model is included as a point of comparison, but is neither theoretically nor empirically supported. Some researchers argued that the two factors of identifying and describing feelings collapse into a single factor (Erni et al., 1997; Kooiman et al., 2002; Loas et al., 1996) since both aspects are highly correlated and several items load significantly on both factors. This would result in a two-dimensional structure for the TAS-20 with a factor for difficulties in dealing with feelings (DIF/DDF) and EOT (Model 2). As a side note, this two-dimensional structure was also the basis of the TAS-R, which then developed into the TAS-20 (Taylor et al., 1992). However, a two-dimensional structure has mainly been found for translated versions in nonpatient samples (Erni et al., 1997; Loas et al., 1996), which might also point to a cultural or translation bias (van de Vijver & He, 2016).

The originally intended three-dimensional structure by Bagby et al. (1994; Model 3a) has been replicated in many studies (Besharat, 2007b; Joukamaa et al., 2001; Meganck et al., 2008; Parker et al., 2003; Parker et al., 2005), although the criteria used to evaluate model fit were sometimes inadequate (e.g., Bressi et al., 1996; Praceres et al., 2012; Seo et al., 2009). This also applies to a recently introduced informant version (Bagby et al., 2021) and many translated versions of the original TAS-20: In 2003, Taylor et al. (2003) conducted a narrative review of the 18 translated versions existing at that time (e.g., a German, Hindi, and Greek version) and concluded that there was “strong support for the generalizability of the three-factor structure of the scale” (p. 281). Since then, 10 more languages have been added (Bagby et al., 2020). Although most studies on the factor structure of the TAS-20 relied on student or healthy adult samples, there is ample evidence that the DIF-DDF-EOT structure can also be found in clinical samples around the world (e.g., Besharat, 2008b; El Abiddine et al., 2017; Loas et al., 2001), but there are exceptions (e.g., Thorberg et al., 2010). Throughout its long history, also the shortcomings of the three-dimensional solution were repeatedly mentioned, for example, the low factor saturation of the EOT factor (e.g., Gignac et al., 2007; Haviland & Reise, 1996).

Besides the originally three-dimensional model, two alternative three-dimensional models have been proposed. In one model (Model 3b), Müller et al. (2003) combined the two factors dealing with feelings (DIF/DDF) and split the EOT factor into two separate factors: Pragmatic thinking (PT) and the lack of (subjective significance or) importance of emotions (IOE). The PT factor captures an unimaginative and utilitarian type of thought processing, originally labeled “pensée opératoire” (Marty & M'Uzan, 1963). The IOE factor deals with “psychological mindedness,” which describes the capacity for self-examination, self-reflection, and introspection. EOT resembles other constructs such as “emotions to facilitate thought” (Mayer et al., 2003) or “emotional utilization” (Austin et al., 2004). In another three-dimensional model (Model 3c, Khosravani et al., 2019; Popp et al., 2008), the factors dealing with feelings still collapse into one, while the remaining items are divided into a core factor of EOT and a factor dealing with flattening of emotions. Even though the labeling of the factors is identical, the allocation of items to factors differs considerably in comparison with the original setup (for more information, see also Figure 1). In general, surprisingly little has been said in the literature about the content of these more nuanced EOT subfactors rendering the assignment mainly data-driven with the risk of capitalizing on chance.

The four-dimensional model (Model 4a; Müller et al., 2003) is a blend of the original and an alternative three-dimensional model (i.e., Model 3a and 3b), in which two factors each describe the handling of feelings (DIF and DDF) and EOT (PT and IOE). This model has received some support besides the original conceptualization (e.g., Craparo et al., 2015; Meganck et al., 2008; Zhu et al., 2003). Sample characteristics such as mean age (Müller et al., 2003), psychiatric status as well as different translations or cultural backgrounds (e.g., Preece, Becerra, Robinson, Dandy, & Allan, 2018) have been discussed as an attempt to explain the inconclusive results, especially for the three- versus the four-dimensional model, but such explanations are often post hoc attempts. Despite the plausible argument that the items map differently in clinical populations than in the general population, most studies showed no differences at all (e.g., Loas et al., 2001; Meganck et al., 2008), although measurement invariance testing in terms of a multigroup confirmatory factor analysis is sparse (for exceptions, see Meganck et al., 2008; Preece, Becerra, Robinson, Dandy, & Allan, 2018).

In several publications a bifactor model was used to describe the TAS-20 (Carnovale et al., 2021; Gignac et al., 2007; González-Arias et al., 2018; Reise et al., 2013; Tuliao et al., 2020; Yun et al., 2019). Besides a general factor that reflects the common variance of all items, specific factors are included to cover additional variance among item sets. The general and the specific factors are uncorrelated with each other. Bifactor models were initially developed in intelligence research (Holzinger & Swineford, 1939), but have recently experienced a renaissance as an important structural representation of multidimensionality beneath a unidimensional construct (Reise, 2012; Reise et al., 2010). In the present case, a general factor of alexithymia (Model 5) represents the target construct, while the nested factors describe specific facets such as EOT. The bifactor is a more flexible version of a high-order factor model in which the formerly first-order factors become the nested factors (Mulaik & Quartetti, 1997) by means of the Schmid–Leiman decomposition (Schmid & Leiman, 1957). The evidence for the appropriateness of the bifactor modeling is mixed, with some studies in favor (e.g., Carnovale et al., 2021; Gignac et al., 2007) and others against (e.g., Tuliao et al., 2020).

A quarter of the TAS-20 items is reversely keyed, that is, high endorsement indicates a low trait level (e.g., “I am able to describe my feelings easily”). To improve model fit, several authors (e.g., Gignac et al., 2007; Meganck et al., 2008; Preece, Becerra, Robinson, Dandy, & Allan, 2018; Tuliao et al., 2020; Watters et al., 2016) suggested specifying a method factor that in addition to the focal dimensions of alexithymia captures a conceptually distinct trait representing a respondent’s response consistency. And indeed, the addition of a method factor usually improved the model fit significantly (e.g., Preece, Becerra, Robinson, Dandy, & Allan, 2018). Such method effects for negatively keyed items are not specific for the TAS, but have repeatedly been reported for many self-report scales (e.g., Gnambs & Schroeders, 2020) and seem to capture a general test-taker’s response style (e.g., DiStefano & Motl, 2006). However, what complicates modeling as a pure method artifact in the case of TAS is that four of the five negatively keyed items load on the EOT factor. Bagby et al. (2007) suggested that the occasionally reported difficulties with this factor may be attributed to item formulation. But Preece, Becerra, Robinson, Dandy, and Allan (2018) showed that this reasoning may not be valid, because the factor loadings were higher on the method factor than on the supposed content factor.

## The Present Study

To address the still ongoing controversy surrounding the structure of the TAS-20, we used meta-analytic structural equation modeling (MASEM; Cheung, 2005a, 2005b; Cheung & Cheung, 2016). MASEM is a two-stage approach: Initially, the correlation coefficients between the item scores are extracted from primary studies, which are subsequently meta-analytically combined into a pooled correlation matrix. Then, confirmatory factor models are fitted to the pooled correlation matrix. Using MASEM, the present research attempts to address three major research questions:

The first question deals with the optimal factor analytic representation of the TAS-20. Although the intended three-dimensional structure has been supported in previous research, it has also been pointed out that past research on the dimensionality of the TAS-20 “has arguably used liberal standards of model fit” (Gignac et al., 2007, p. 254). In addition, alternative factor structures were only in part systematically investigated (e.g., Preece, Becerra, Robinson, Dandy, & Allan, 2018). With some exceptions (e.g., Bagby et al., 1994), the examinations relied on selective and small samples. In contrast, MASEM provides the opportunity to combine and weight the mixed previous findings in the literature, so that more robust statements about the dimensionality of the TAS-20 can be made beyond a specific sample. The confirmatory approach of MASEM also provides the opportunity to empirically test the original conceptualization of the construct alexithymia. Once Kurt Lewin (1952, p. 169) aptly wrote “there is nothing more practical than a good theory.” Taking this bon mot seriously, the question of the dimensionality of the TAS-20 and the theoretical assumptions that led to its construction, also have implications for clinical practice and should promote further research. Either the intended structure is replicated, supporting the original conceptualization of the construct and strengthening the use of the TAS-20 as a standard instrument for the assessment of alexithymia. Or, in case the assumed dimensionality is not empirically supported, the measure has to be refined or, even more fundamentally, the theoretical conceptualization of alexithymia must be revised.

A related question is whether the psychometric quality of the measurement instrument can be improved by introducing additional method factors such as an orthogonal method factor for the reversely coded items. Is the ambiguous status of the EOT factor (i.e., split into two factors and low factor saturation in general) related to the unbalanced distribution of reversely coded items per factor? The psychometric aspects are also significant from a practical point of view because the correct modeling reflects on how to best derive person estimates that enable a valid clinical assessment (see also Reise et al., 2013). The magnitude of factor correlations can also indicate if it is appropriate to rely on a sum score for the complete alexithymia scale.

Finally, we examine measurement invariance across potential sources of heterogeneity in the meta-analytic results. Since the TAS-20 has been often studied in nonclinical samples, the question arises whether the factor structure between patients and nonpatients varies. Early on, questions arose about the comparability of the instrument depending on the psychiatric status. For example, Haviland and Reise (1996) reported that a three-dimensional model fitted best in a psychiatric and a student sample, but the allocation of items to factors largely differed resulting in different interpretations of the factors. With respect to measurement invariance of the TAS-20 across translations, a narrative review of 19 studies concluded that there was “strong support for the generalizability of the three-factor structure of the scale” across languages and cultures (Taylor et al., 2003, p. 281). However, only fit indices concerning the three-factor structure were compared without systematically studying alternative models. Thus, we study bias at the item level that might be introduced to the measurement by adapting the measure to other languages and cultures.

## Method

### Meta-Analytic Data Base and Coding

**Figure 2.**
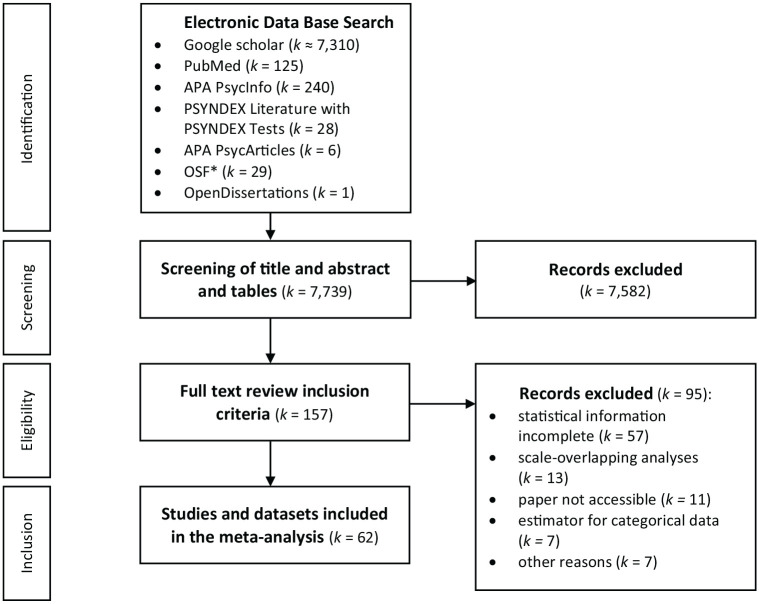
Overview of the Literature Search Process. *Note.* The search term was a Boolean expression: (TAS-20 OR “Toronto Alexithymia Scale”) AND (“factor analysis” OR “factor structure” OR “principal component analysis” OR “item analysis”). For more detailed information on the reasons for exclusion see screening_studies.xlsx in the online repository. ^*^For screening the open science framework repository the search term was reduced to TAS-20 OR “Toronto Alexithymia Scale.”

The search for primary studies reporting on the factor structure of the TAS-20 included major scientific databases (PubMed, PsycArticles, PsycINFO, and PSYNDEX), data repositories of the open science framework (OSF), and Google Scholar. In December 2020, we identified 7,739 potentially relevant journal articles and data archives using the Boolean expression (TAS-20 OR “Toronto Alexithymia Scale”) AND (“factor analysis” OR “factor structure” OR “principal component analysis” OR “item analysis”). Additional studies were derived from the references of all identified articles (“rolling snowball method”). After reviewing the titles, abstracts, and, subsequently, tables of these manuscripts, we made a full-text review of 157 studies. We retained all studies that met the following criteria: (a) In the study the original (or a translated version) of the TAS-20 was administered (i.e., all studies that used the TAS-26 or TAS-23 were excluded, despite an overlap in items; informant reports were also not considered), (b) the publication (or data) was published since the initial publication by Parker et al. (1993)^1^, and (c) the necessary item-level statistics were available including the loading pattern from an exploratory (or confirmatory) factor analysis, the full covariance (or correlation) matrix, or raw data. If oblique factor rotations were used, we only considered studies that also reported the respective factor correlations. In case the raw data of a study was available, we calculated the respective covariance matrix. Moreover, we excluded studies that reported the results of a factor analysis that was jointly conducted with items of another measure besides the TAS-20. This literature search and screening process resulted in 62 studies with 88 samples, which could be included in our meta-analysis (see Figure 2, for an overview).

We defined all relevant information to be extracted from each publication accompanied with relevant coding guidelines (e.g., response format) in a coding protocol, which is accessible in the online supplement. The focal information pertained to factor loading patterns and correlation matrices of the 20 items included in the TAS-20. If different factor solutions for one and the same sample were available, we used the factor loading pattern with the highest number of factors. In addition, descriptive information was collected on the sample (e.g., sample size, country, mean age, percentage of female participants), the publication (e.g., publication year), and the reported factor analysis (e.g., factor analytic method, type of rotation). All studies were coded by the first author. To evaluate the coding process three-quarters of the studies were independently coded a second time by the second author. Intercoder agreement was quantified using Krippendorff’s alpha (Krippendorff, 2013), which indicate good agreement for values larger than .80. Overall the intercoder agreement was excellent with values for study characteristics ≥.90 and for the factor analytic results of the primary studies of .98.

### MASEM Procedure

We examined the factor structure of the TAS-20 with MASEM, which is the integration of two techniques with a long-standing tradition, but with limited exchange between both disciplines—meta-analysis and structural equation modeling (Cheung, 2013; Cheung & Chan, 2009; Jak, 2015). In the first step of MASEM, the item-level correlation matrices were pooled using a fixed-effects meta-analysis,^2^ because simply taking a pooled correlation matrix as input for a structural equation model is inaccurate (see Cheung & Chan, 2005a, for a full account). In more detail, we used the zero-order Pearson product-moment correlations between the items of the TAS-20 as effect size measures (for a graphical representation of the correlation matrix of all correlation matrices, see online supplement, Figure OS 1). Three studies provided the raw data in an online repository. Most studies, however, reported the factor pattern matrices from exploratory (27 samples in 25 studies) or confirmatory factor analysis (58 samples in 35 studies). Following Gnambs and Staufenbiel (2016), we calculated the model-implied item-level correlations based on the reported factor pattern matrices. Few studies neglected to report the full factor loading pattern and excluded small loadings falling below .40. In this case, a value of zero was imputed for the missing factor loadings, because Monte Carlo simulations indicated that this approach results in unbiased estimates of meta-analytic factor patterns (Gnambs & Staufenbiel, 2016).

In the second step of MASEM, the derived pooled correlation matrix was subjected to factor analytic models. We first report the results of an exploratory factor analysis (EFA). Following the recommendations of Auerswald and Moshagen (2019), we used several criteria to decide on the number of factors to retain, including Velicer’s (1976) minimum average partial (MAP) test, Horn’s (1965) parallel analysis, Bayesian information criteria (BIC), and sequential χ^2^ model tests. Moreover, competing measurement models were tested using confirmatory factor analysis with a weighted least square estimator using the asymptotic variance–covariance matrix of the pooled correlations from the first step as weights (Cheung & Chan, 2005a). In line with conventional standards (see Schermelleh-Engel et al., 2003), we used the following cutoff criteria: Comparative fit index (CFI) ≥ .95, root mean square error of approximation (RMSEA) ≤ .08, and a standardized root mean square residual (SRMR) ≤ .10 were interpreted as “acceptable” and models with CFI ≥ .97, RMSEA ≤ .05, and SRMR ≤ .05 as “good” fit.

### Software and Open Practices

The exploratory factor analyses were conducted using the psych package version 2.0.12 (Revelle, 2020). Confirmatory factor analyses and pooling correlations were done with the R package metaSEM (version 1.2.5; Cheung, 2020), which relies on OpenMx (version 2.18.1, Neale et al., 2016). To promote transparency and reproducibility of our analyses, all coded data and analyses scripts are provided in an online repository at https://osf.io/uxtks/.

## Results

### Study Characteristics

The meta-analysis included 88 samples nested in 62 studies that were published between 1994 and 2020. Median sample size was *Mdn* = 327 participants (total *N* = 69,722; *Min* = 99; *Max* = 12,706) with approximately 54.4% women and a reported mean age of 29.2 years (*SD* = 10.0). Because the TAS-20 has been translated in many different languages (Bagby et al., 2020), the present meta-analysis included data from 25 different countries, with most samples coming from Canada (20.5%), the United States (10.2%), Australia, Germany, and Iran (each 6.8%). The most frequently used translated versions (with parentheses giving the number of samples) were in French (8), German (7), Farsi (6), Portuguese (5), and Japanese (5), for which measurement invariance to the original English version (34) will be considered in more detail. The complete list of languages covered in this meta-analysis also includes Turkish (4), Spanish (4), Korean (3), Dutch (3), Italian (2), Finnish (2), Chinese (2)^3^, Swedish (1), Hindi (1), and Arabic (1). Three-quarters of the samples were nonclinical (*k* = 66), consisting of mainly undergraduate university students. Twenty samples were clinical-psychiatric samples of a wide spectrum (e.g., somatoform disorder, anxiety, substance use disorders). The characteristics of all samples are given in the coding sheet in the online repository.

### Exploratory Factor Analyses

**Figure 3.**
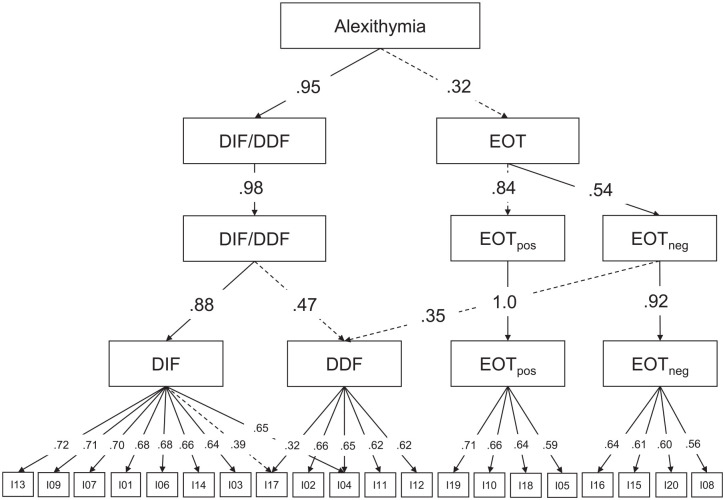
Bass-ackward analyses of the pooled correlation matrix. *Note. N* = 69,722. Varimax-rotated components were derived from the pooled correlation matrix. Correlations below .30 were omitted and below .50 were shown as dashed lines. DIF = difficulty identifying feelings, DDF = difficulty describing feelings, EOT = external-oriented thinking.

The different criteria that can be used to determine the number of factors in EFA (Auerswald & Moshagen, 2019; Ruscio & Roche, 2012) came to rather different conclusions: The MAP procedure suggested two factors, Horn’s parallel analysis four factors, the sample-size adjusted BIC achieved a minimum with five factors, and the sequential χ² model test recommended extracting six factors. This heterogeneity may indicate that these approaches were not designed for such large sample sizes, that is, depending on the criterion even negligible improvements of consecutive factor solutions were considered significant. Instead of presenting a single factor solution, we offer the results of bass-ackwards analyses (Goldberg, 2006) to better understand the unfolding of the hierarchical structure of the TAS-20 (see Figure 3). This method has been mostly applied in personality research (e.g., Wright et al., 2012) and involves the estimation of a series of orthogonal principal component analyses with an increasing number of components. Please note that only orthogonal rotations produce interpretable between-level factor score correlations, which can inform about the structure of the TAS-20 at different levels of abstraction. The one-component solution explained only 26% of the total variance, and seven items had a factor loading below .30, indicating distinct facets of alexithymia. The first important distinction concerned dealing with feelings (DIF/DDF) and thinking style (EOT). At the third level, the EOT component was split up into positively and negatively keyed items. Only at the last level given in Figure 3, the broad DIF/DDF component is separated into DIFs and DDFs. In summary, three central findings can be extracted from the bass-ackwards analyses: (a) alexithymia is a heterogeneous, multifaceted construct, (b) the EOT facet breaks down into equal parts of positively and negatively keyed items which is not paralleled by a content-based distinction, and (c) the two facets DIF/DDF are substantially related and might even collapse.

### Confirmatory Factor Analyses

To compare the different theoretical ideas concerning the structure of the TAS-20, we estimated nine measurement models (model fits are given in Table 1, all parameter estimates are provided in the online supplement, Table OS 1). The confirmatory factor analyses showed that the original three-dimensional model proposed by Parker et al. (1993) and the four-dimensional model which has also received substantial support in the literature (e.g., Gignac et al., 2007; Meganck et al., 2008) fitted the data best, although the CFI was below the threshold of .95. The difference between the two models is that the factor for EOT is split into the two facets PT and IOE. Comparing both models, there was strong evidence for keeping the original model, because it is more parsimonious and the correlation between EOT facets was very high (*r* = .94), which is in line with previous research (Meganck et al., 2008). The correlation between the factors of the three-dimensional solution was highest between DDF and DIF (*r* = .77), and moderate for the correlations with the EOT factor (*r*_DDF/EOT_ = .47 and *r*_DIF/EOT_ = .32). The reliability estimates (i.e., ω according to McDonald, 1999) were ω_DIF_ = .84, ω_DDF_ = .75, and ω_EOT_ = .62. The split of the EOT factor was not accompanied by an improvement in reliability due to the small size of the item sets (ω_IOE_ = .56 and ω_PT_ = .31). All other measurement models were not sufficiently supported by the empirical data. This also applied to the bifactor model. Although the model fit indices indicated good fit, the pattern of factor loadings was problematic with many low factor loadings on the general factor, close-to-zero factor loadings on the specific DDF^#^ factor,^4^ and larger loadings on the EOT^#^ factor than on the general factor. Because simulation studies showed that low factor loadings invalidate traditional cutoffs used to evaluate model fit (Heene et al., 2011), the bifactor model does not adequately capture the dimensionality of the TAS-20. Such anomalous results of bifactor models are not uncommon and have led to the proposal of alternative bifactor representations with a reference factor (Eid et al., 2017). However, analyses with DDF as a reference factor did not rectify the problem of several low factor loadings (see Model 5b in Table OS 1 in the online supplement).

**Table 1.** Fit Statistics for Different Confirmatory Factor Models of the TAS-20.

No	Model	χ^2^	*df*	CFI	RMSEA	SRMR	AIC	BIC
1	Unidimensional model (Alex)	29,912.4	170	.768	.050^b^	.084	29572.4	28016.5
2	Two-dimensional model (DIF/DDF-EOT)	16,757.7	169	.871	.038^b^	.056	16419.7	14873.0
3a	Original three-dimensional model (DIF-DDF-EOT)	8,424.2	167	.936	.027 [.026, .027]	.041	8090.2	6561.8
3b	Alternative three-dimensional model (DIF/DDF-PT-IOE)	16,635.4	167	.872	.038^b^	.055	16301.4	14772.9
3c	Alternative three-dimensional model (DIF/DDF-EOT-IOE)^a^	22,216.9	167	.828	.044^b^	.073	21882.9	20354.4
4	Four-dimensional model (DIF-DDF-PT-IOE)	8,274.7	164	.937	.027 [.026, .027]	.040	7946.7	6445.8
5	Bifactor model with three original factors as nested factors	6,159.8	150	.953	.024 [.023, .024]	.026	5859.8	4487.0
6	Original three-dimensional model + nested method factor	1,756.5	162	.988	.012 [.011, .012]	.013	1432.5	−50.2
7	Original three-dimensional model + correlated residuals	1,753.9	157	.988	.012 [.012, .013]	.013	1439.9	3.0

*Note. N* = 69,722. *k* = 88. A version of this table for the samples using the English version only can be found in the online supplement (Table OS 2). TAS-20 = Toronto Alexithymia Scale; DIF/DDF = difficulty identifying and describing feelings; DIF = difficulty identifying feelings; DDF = difficulty describing feelings; EOT = external-oriented thinking; PT = pragmatic thinking; IOE = lack of (subjective significance or) importance of emotions. CFI = comparative fit index; *df* = degrees of freedom; SRMR = standardized root mean square residual; RMSEA = root mean square error of approximation; AIC = Akaike information criterion; BIC = Bayesian information criterion.

a In this solution, the factor labels were retained, although the items load on other factors compared to the standard solution. ^b^Confidence interval could not be computed.

The TAS-20 includes a total of five negatively keyed items, of which four load on the EOT factor. A model that—in addition to the basic three-dimensional structure—introduced a nested method factor that captures that method-specific variance associated with the reverse item wording yielded a significant improvement in model fit (see Table 1). In the psychometric literature on the TAS-20, it has been reported that such a method factor model might be superior to other models (Meganck et al., 2008; Preece et al., 2021; for a more critical evaluation, see Gignac et al., 2007). However, taking a closer look at the loading pattern, we noticed that all negatively keyed EOT items loaded higher on their method factor than on the actual content factor. This pattern is due to the fact that half of the EOT items are negatively keyed, so that a clear instantiation of the factor is lacking. Differently put, the factor that was designed as a method factor also captured content variance, which blurs a clear separation between both sources of variance. As a last model, we estimated a model that included the three facets of alexithymia as well as residual correlations between all negatively keyed items. Such a model is also known as *correlated trait correlated uniqueness model* (Marsh & Grayson, 1995) and fitted the data well in terms of CFI and RMSEA (see Table 1). Residual correlations varied in the typical low range between .08 and .26 (*Mdn* = .16). However, even with this type of modeling, the factor loadings of the negatively keyed items on the EOT factor were rather small. Taken together, the results of the confirmatory factor models showed that the originally proposed three-factor structure provided the most consistent representation of the TAS-20 in terms of model fit, factor loading pattern, and factor correlations.

### Measurement Invariance Testing

The TAS-20 has been translated into more than two dozen different languages including Arabic, Hebrew, and Mandarin (for an overview, see Bagby et al., 2020; Parker et al., 2003). In this meta-analysis, data from 15 languages (besides English) were included. In retrospect, it is striking that many of the alternative measurement models were proposed for other language versions, raising the question of whether systematic bias is introduced through translation or a different cultural context. To address this question, we estimated a multigroup MASEM for six language groups (Cheung & Chan, 2005b; Jak & Cheung, 2018). We included all languages for which at least five samples were available: English (34), French (8), German (7), Farsi (6), Portuguese (5), and Japanese (5). While studies administering the French or Farsi versions of the TAS-20 each used the same translation, most non-English language versions were based on different translations: For Portuguese and Japanese two different translations were available and in the German samples even three slightly different translations were administered—as far as this can be deduced from the available information and references. For most studies, however, the exact wording of the items was not available.

**Figure 4.**
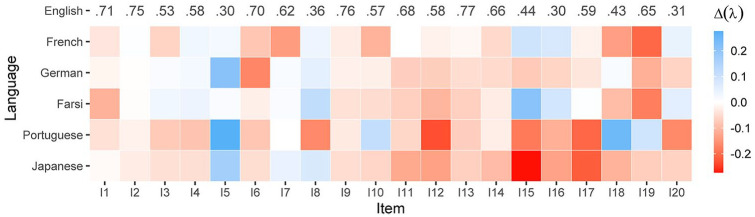
Differences in factor loadings between the English and five translated versions. *Note*. Reference is the original English version. Values in the first row are the standardized factor loadings of the three-factor model of the English version.

Table 2 shows the model fit of the original three-dimensional model for all language versions (ordered by the number of samples included). With a CFI close to .95 the English version (*k* = 34 samples) yielded a significantly better fit than analyses including all samples. In terms of model fit, the Farsi and the Portuguese version had an excellent fit, while all other language versions exhibited considerable misfit (see Table 2). Especially, the German version was not in line with the theoretical assumptions. Figure 4 shows the difference in the factor loadings between the original three-dimensional model for the English version (values in the first row) and its translated counterparts. Larger factor loadings in translated versions were erratic and rare, except for Item 5 (“I prefer to analyze problems rather than just describe them.”) that performed better in most of the translated versions, *M*(Δλ_5_) = .14. Smaller factor loadings were more common and for some translations substantial, especially when considering the absolute level of the factor loadings. These differences were most pronounced for Item 12 (“People tell me to describe my feelings more.” *M*(Δλ_12_) = −.11). Across the language versions, the mean absolute change in factor loadings was mainly small (see also second last column in Table 2). Taken together and bearing in mind the small number of samples included in the calculations, the original English and the translated Farsi and Portuguese versions yielded satisfactory results in terms of model fit. In contrast, the French, German, and Japanese versions deviated considerably from the English version. This finding is in line with Fukunishi et al. (1997), who reported on low factor correlations of the EOT factor for the Japanese version (see also ω_EOT_ in Table 2). For German, there were at least three slightly different versions which might have contributed to the fact that for the German translations in particular alternative factor models have been proposed (Franz et al., 2001; Koch et al., 2015; Müller et al., 2003; Popp et al., 2008). For the French version, a previous study reported on strict measurement invariance across languages (Watters et al., 2016). But, the overall fit even of the configural model was unsatisfactory and, moreover, the invariance testing procedure was not correctly specified (see also Schroeders & Gnambs, 2020).

**Table 2.** Comparison of Model Fit, Reliability Estimates, and Factor Loadings Across Languages.

Language	*k*	*n*	χ^2^	*df*	CFI	RMSEA	SRMR	ω			Δ(λ)	
								DIF	DDF	EOT	|*M*|	[Min, max]
English	34	28,514	3378.8	167	.949	.026 [.025, .027]	.046	.86	.77	.64	—	—
French	8	10,721	2635.7	167	.881	.037 [.036, .038]	.061	.82	.77	.60	.06	[−.21, .09]
German	7	3,692	2556.1	167	.740	.062 [.060, .064]	.127	.83	.74	.63	.06	[−.17, .21]
Farsi	6	2,384	9.8	167	1.00	.000 [.000, .000]	.004	.83	.75	.66	.07	[−.18, .21]
Portuguese	5	2,122	213.7	167	.988	.011 [.006, .016]	.030	.83	.65	.67	.11	[−.23, .28]
Japanese	5	6,078	1220.1	167	.903	.032 [.031, .034]	.048	.83	.66	.55	.09	[−.27, .17]

*Note*. Values are based on the originally proposed three-dimensional model. *k* = number of samples; CFI = Comparative Fit Index; RMSEA = Root Mean Square Error of Approximation; SRMR = Standardized Root Mean Square Residual; ω = McDonald’s omega; DIF = difficulty identifying feelings; DDF = difficulty describing feelings; EOT = external-oriented thinking; Δ(λ) = difference in the standardized factor loadings of the translated version in reference to the English version; |*M*| = mean of absolute differences in factor loadings between the English and the translated version.

We also report the measurement invariance testing across psychiatric status. Because language or culture has a clear influence on the dimensional structure of the TAS-20, we constrained our analytical sample to the subset that used the English version (*k* = 34). Model fit was satisfactory in the nonpsychiatric sample (*k =* 29, *n* = 27,453, χ^2^ = 3,368.8, *df* = 167, CFI = .948, RMSEA = .026, SRMR = .048) and excellent in the patient sample (*k* = 5, *n* = 1,061, χ^2^ = 88.0, *df* = 167, CFI = 1.00, RMSEA = .000, SRMR = .026). A comparison of the factor loadings between patients and nonpatients showed mainly small differences (for all parameter estimates, see Table OS 3 in the online supplement)—except for Item 10, which belongs to the factor EOT (“Being in touch with my feelings is essential,” *M*(Δλ_10_) = .22 in favor of the clinical sample).

## Discussion

Given the vast research literature on the dimensionality of TAS-20 and the alternative models that have been proposed, the present meta-analysis attempted to give an evidence-based verdict on the internal structure of the TAS-20: In line with its theoretical ideas (Bagby et al., 1994, 2020; Taylor & Bagby, 2020), the original three-dimensional structure describes the available data best. In contrast to the “liberal standards of model fit” (Gignac et al., 2007, p. 254) that have been used previously, at least for the English version meta-analytic evidence pointed to a good model fit for the original representation. This can also be seen as strong support for the original theory-driven conceptualization of the TAS-20. The alternative solutions that have been discussed try to substantiate an empirically found factor structure. On the one hand, these models proposed collapsing the factors that describe the handling of feelings and, on the other hand, splitting the more heterogeneous EOT factor, did not provide substantial improvements. Such post hoc adjustments might lead to a better model fit in a specific data set, but these solutions are seldom stable across different studies and samples. With this in mind, the theory-informed and confirmed three-dimensional structure of the TAS-20 is preferable over others. The results of the bass-ackwards analysis indicated that these alternative solutions blend into each other at different levels of granularity. Thus, the seemingly competing models for the dimensionality of the TAS-20 found in individual studies are better understood from a hierarchical perspective. Since the median of the samples’ sizes included in the present manuscript was 327 such diverging factor solutions might simply occur by chance because factor loadings tend to be highly unstable in smaller samples (e.g., *N* < 500; Hirschfeld et al., 2014). Moreover, factor solutions might also depend on sample characteristics such as age, educational level, or psychiatric disorder. In particular, the usefulness of the negatively keyed items was questioned in clinical samples. Kojima et al. (2001) speculated that the splitting between positively and negatively keyed items within the EOT is due to the more complex wording of the items and the lower mental flexibility of the alexithymic patients. And Ryder et al. (2018) pointed out that the EOT items, in comparison with the remaining items, emphasize interests and preferences rather than deficits. In this context, it is also noteworthy that all four negatively keyed EOT items require a decision between two evaluation objects.

For the more advanced models that try to tap method-specific variance, the results were more surprising. The models in which systematic method variance arising from the negatively keyed items were addressed lead to problematic factor loadings. This notion held for the nested factor model with a factor that is orthogonal to the trait factor as well as for the correlated-trait-correlated-uniqueness model. Thus, the intended separation between method and trait variance could not be achieved, which highlights two design flaws that have been frequently addressed (Gignac et al., 2007; Reise et al., 2013) and that prevent a more adequate modeling: First, half of the EOT items are negatively keyed, thus lacking a prototypical instantiation. Second, four of the five negatively keyed items belong to one and the same factor which confounds method-related and content-related variance. Therefore, some studies rephrased the negatively into positively keyed items, however, without arriving at a clearer factor solution (e.g., Maggini & Raballo, 2004). Also, the bifactor model which assumes a general factor of alexithymia and uncorrelated specific factors for the facets was problematic resulting in several low factor loadings. This corroborates recent modeling attempts which also resulted in low or even negative factor loadings (González-Arias et al., 2018; Tuliao et al., 2020). We agree with Reise et al. (2013, p. 138) that fitting a highly restricted multidimensional model such as the bifactor model for the TAS-20 “seems a tedious exercise.” From a practical point of view, the three-dimensional structure with clearly separable factors indicate that the subscale scores provide a more adequate picture of alexithymia than a general score. Whether this superiority in model fit also translates into visible, clinical consequence has to be subject of further clinical research. The total scale score is more reliable since it is based on more items, but it is assumed that the subscale scores might have added value (cf. Sekely et al., 2018). If a more fine-grained feedback really offers an increment (e.g., detecting an improvement of specific symptoms in the course of a treatment), is a question, that cannot solely be answered based on measurement models.

The subsequent measurement invariance tests showed that part of the overall model misfit of the three-dimensional solution stemmed from translated versions. It should be noted that the confirmatory way of testing the comparability across languages, is much stricter than Tucker’s congruence coefficient (Lorenzo-Seva & ten Berge, 2006), which is a simpler descriptive gauging the similarity of correlation matrices. It is somewhat ironic that one of the strengths of the instrument, that is, the global spread of the TAS-20, is also one of its weaknesses, since the translated versions seem to contribute to biases in the dimensional structure. Different types of bias in cross-cultural assessment might be responsible for these translation biases (van de Vijver & Tanzer, 2004). For example, item bias (often expressed as differential item functioning) might occur if a poor translation causes a shift in the items’ content or if culture-specific interpretations of the item content exist (Chen, 2008). This issue could be tackled with proper translation and adaption techniques (Geisinger & McCormick, 2012). A more serious problem for measuring alexithymia across cultures might be construct bias, that is, differences across cultures in the construct itself. For example, Kirmayer (1987) argued that alexithymia is often understood as an intrapsychic process or deficit, but that this emphasis might obscure the impact of the social and cultural context. Thus, cultural display rules affect the readiness or intensity with which more collectivistic cultures such as the Japanese show their feelings openly and social norms shape what is considered appropriate behavior (Fukunishi et al., 1997; Matsumoto et al., 2008). In this context, it is plausible that Item 15, “I prefer talking to people about their daily activities rather than their feelings,” is heavily affected by the sociocultural context. As Ryder et al. (2018, p. 41) pointed out, the EOT items that emphasize interests and preferences rather than deficits, might “reflect a cognitive style rooted in cultural values about emotion.” In retrospect, one has to admit that alexithymia as assessed by the TAS-20 has “evolved from the clinical observation made on populations in North America and Western Europe” (Fukunishi et al., 1997, p. 797). Thus, in other cultural contexts alexithymia might not manifest in the same manner. With respect to measurement invariance in patient versus nonpatient samples, our meta-analytic results are reassuring. Model fit in the clinical samples excelled the one in the nonclinical samples. But, it should be critically noted that these estimates were based on a comparatively small and heterogeneous sample.

### Limitations and Future Directions

Some limitations of the present meta-analysis have to be taken into account: First, the recovery of population factors in individual studies can be impeded by sampling error (MacCallum et al., 2001) or highly skewed response distributions (with many people reporting no symptoms; Gaskin et al., 2017). Although pooling results across diverse samples should provide more robust inferences on the population factor structure, we were unable to systematically examine the distributions of the included studies. However, comparisons between clinical and nonclinical samples showed highly comparable measurement models for the TAS-20 and, thus, indicated robust results across heterogeneous populations. Second, the factor analyses relied on sample statistics that, for the most part, were reproduced from reported loading structures. Compared with other MASEMs of psychological measurement instruments such as Rosenberg’s Self-Esteem Scale (Gnambs et al., 2018) or the short version of the General Health Questionnaire (Gnambs & Staufenbiel, 2018), for the TAS-20 only few studies provided the raw data to compute these matrices, probably because in clinical settings legal restrictions or ethical considerations prevent data sharing. Therefore, we could not compare the stability of the factor structure across different data sources. Although simulation research (Gnambs & Staufenbiel, 2016) and empirical comparisons using other instruments demonstrated the validity of the adopted MASEM approach, future studies are encouraged to cross-validate the presented findings with independent raw data, preferably, from large-scale, representative samples. Moreover, *Community-Augmented Meta-Analyses* (Burgard et al., 2021), which combine an open repository for meta-analytic data with meta-analytic analysis tools, might be an effective way to circumvent data sharing issues and develop a continually updating database providing up-to-date information on the measurement properties of the TAS-20.

In addition to the findings reported in this article, the MASEM results might serve as a starting point for future research and further refinement of the TAS-20. For example, a 20-item questionnaire might be brief, but still too extensive for large-scale studies that rely on strongly abbreviated versions. The data at hand (i.e., the weighted correlation matrix) might allow compiling a short version of the TAS-20 (see also Williams & Gotham, 2021). Analyzing the meta-analytic results with modern item selection algorithms such as *Ant Colony Optimization* (Schroeders et al., 2016) several psychometric criteria could be considered simultaneously. For example, an abbreviated version with a sound measurement model and reliable factors could be derived that also approximate the relations to covariates or ensure measurement invariance across cultures (see also Jankowsky et al., 2020). The focus of the present meta-analysis was on the internal structure of the TAS-20. It would be worthwhile to extend the analysis with an additional meta-analytic investigation studying the relation of the TAS-20 to relevant constructs (e.g., mentalizing, empathy), thus, to place the measure into a larger nomological network.

Alexithymia is and likely will be an influential construct in clinical and nonclinical research and practice. The prevalent measure of alexithymia, the TAS-20, has previously attracted various psychometric criticisms. In the present meta-analysis, we examined the factorial structure across diverse samples and translations. Overall, these analyses corroborated the hypothesized three-factor structure representing DIF, DDF, and EOT. However, weaknesses in the construction of various translated versions of the TAS-20 might impede cross-cultural research on alexithymia. Although the further development of the TAS series (Bagby et al., 2020; Taylor et al., 2020) seems to reconnect to the original clinical observations and theoretical ideas by including the reduced fantasizing as a component of the “imaginal process” (for an opposing opinion, see Preece, Becerra, Robinson, et al., 2020), we believe that the TAS-20 is an important milestone, which will continue to serve as a reference in the assessment of alexithymia.

## Supplemental Material

sj-pdf-1-asm-10.1177_10731911211033894 – Supplemental material for The Structure of the Toronto Alexithymia Scale (TAS-20): A Meta-Analytic Confirmatory Factor Analysis Click here for additional data file. Supplemental material, sj-pdf-1-asm-10.1177_10731911211033894 for The Structure of the Toronto Alexithymia Scale (TAS-20): A Meta-Analytic Confirmatory Factor Analysis by Ulrich Schroeders, Fiona Kubera and Timo Gnambs in Assessment

## Notes

1. Non-English articles were translated if necessary, using online translation tools.

2. We used a fixed-effects model instead of random-effect because (a) the latter had estimation problems due to the complexity of the model and (b) the empirical between-study heterogeneity was low in the present study (van Erp et al., 2017).

3. Chinese was used as a label for a Mandarin version (with simplified characters) and a Cantonese version (with complex characters), respectively.

4. Please note that factors with # as superscript are associated with different interpretations across models although they have the same label.

## References

[bibr1-10731911211033894] *Aluja A. Malas O. Urieta P. Worner F. Balada F. (2020). Biological correlates of the Toronto Alexithymia Scale (TAS-20) in cardiovascular disease and healthy community subjects. Physiology & Behavior, 227(December), Article 113151. 10.1016/j.physbeh.2020.113151 32841673

[bibr2-10731911211033894] *Anderson S. (2003). A stress-emotion skill model of adaptation: A study in the rubric of emotional intelligence [Doctoral dissertation, University of Wollongong]. https://ro.uow.edu.au/theses/2124/

[bibr3-10731911211033894] Apfel R. J. Sifneos P. E. (1979). Alexithymia: Concept and measurement. Psychotherapy and Psychosomatics, 32(1-4), 180-190. 10.1159/000287386 550171

[bibr4-10731911211033894] Auerswald M. Moshagen M. (2019). How to determine the number of factors to retain in exploratory factor analysis: A comparison of extraction methods under realistic conditions. Psychological Methods, 24(4), 468-491. 10.1037/met0000200 30667242

[bibr5-10731911211033894] Austin E. J. Saklofske D. H. Huang S. H. S. McKenney D. (2004). Measurement of emotional intelligence: Testing and cross-validating a modified version of Schutte et al.’s (1998) measure. Personality and Individual Differences, 36(3), 555-562. 10.1016/S0191-8869(03)00114-4

[bibr6-10731911211033894] *Ayranci E. (2014). Effects of emotion recognition and alexithymia on motivation to lead: Evidence from Turkey. International Journal of Academic Research in Accounting, Finance and Management Sciences, 4(2), 105-116. 10.6007/IJARAFMS/v4-i2/817

[bibr7-10731911211033894] Bagby R. M. Parker J. D. Onno K. A. Mortezaei A. Taylor G. J. (2021). Development and psychometric evaluation of an informant form of the 20-item Toronto Alexithymia Scale. Journal of Psychosomatic Research, 141(February), Article 110329. 10.1016/j.jpsychores.2020.110329 33316631

[bibr8-10731911211033894] *Bagby R. M. Parker J. D. A. Taylor G. J. (1994). The twenty-item Toronto Alexithymia scale–I: Item selection and cross-validation of the factor structure. Journal of Psychosomatic Research, 38(1), 23-32. 10.1016/0022-3999(94)90005-1 8126686

[bibr9-10731911211033894] Bagby R. M. Parker J. D. A. Taylor G. J. (2020). Twenty-five years with the 20-item Toronto Alexithymia Scale. Journal of Psychosomatic Research, 131(April), Article 109940. 10.1016/j.jpsychores.2020.109940 32007790

[bibr10-10731911211033894] Bagby R. M. Taylor G. J. Quilty L. C. Parker J. D. A. (2007). Reexamining the factor structure of the 20-item Toronto Alexithymia Scale: Commentary on Gignac, Palmer, and Stough. Journal of Personality Assessment, 89(3), 258-264. 10.1080/00223890701629771 18001226

[bibr11-10731911211033894] Bagby R. M. Taylor G. J. Ryan D. (1986). Toronto Alexithymia Scale: Relationship with personality and psychopathology measures. Psychotherapy and Psychosomatics, 45(4), 207-215. 10.1159/000287950 3588819

[bibr12-10731911211033894] *Besharat M. A. (2007a). Psychometric properties of Farsi version of the Toronto Alexithymia Scale-20 (FTAS-20). International Journal of Psychology, 1(1), 64-79.

[bibr13-10731911211033894] *Besharat M. A. (2007b). Reliability and factorial validity of a Farsi version of the 20-item Toronto Alexithymia Scale with a sample of Iranian students. Psychological Reports, 101(1), 209-220. 10.2466/pr0.101.1.209-220 17958129

[bibr14-10731911211033894] *Besharat M. A. (2008a). Assessing reliability and validity of the Farsi version of the Toronto Alexithymia Scale in a sample of substance-using patients. Psychological Reports, 102(1), 259-270. 10.2466/pr0.102.1.259-270 18481685

[bibr15-10731911211033894] *Besharat M. A. (2008b). Psychometric characteristics of Persian version of the Toronto Alexithymia Scale-20 in clinical and non-clinical samples. Iranian Journal of Medical Sciences, 33(1), 1-6. https://ijms.sums.ac.ir/article_39805.html

[bibr16-10731911211033894] *Bolat N. Yavuz M. Eliaçik K. Zorlu A. Evren C. Köse S. (2017). Psychometric properties of the 20-item Toronto Alexithymia Scale in a Turkish adolescent sample. Anatolian Journal of Psychiatry, 18(4), 362-368. 10.5455/apd.239284

[bibr17-10731911211033894] *Boutemy G. C. (2000). Alexithymia as related to the use of language and symptom reporting [Master's thesis, The College of William & Mary]. William & Mary Digital Archive. 10.21220/s2-jhtx-r704

[bibr18-10731911211033894] *Bressi C. Taylor G. J. Parker J. D. A. Bressi S. Brambilla V. Aguglia E. Allegranti I. Bongiorno A. Giberti F. Bucca M. Todarello O. Callegari C. Vender S. Gala C. Invernizzi G. (1996). Cross validation of the factor structure of the 20-item Toronto Alexithymia Scale: An Italian multicenter study. Journal of Psychosomatic Research, 41(6), 551-559. 10.1016/S0022-3999(96)00228-0 9032718

[bibr19-10731911211033894] *Briganti G. Linkowski P. (2019). Network approach to items and domains from the Toronto Alexithymia Scale. Psychological Reports, 123(5), 2038-2052. 10.1177/0033294119889586 31752608

[bibr20-10731911211033894] Burgard T. Bošnjak M. Studtrucker R. (2021). Community-augmented meta-analyses (CAMAs) in psychology: Potentials and current systems. Zeitschrift Für Psychologie, 229(1), 15-23. 10.1027/2151-2604/a000431

[bibr21-10731911211033894] Carnovale M. Taylor G. J. Parker J. D. A. Sanches M. Bagby R. M. (2021). A bifactor analysis of the 20-item Toronto Alexithymia Scale: Further support for a general alexithymia factor. Psychological Assessment, 33(7), 619-628. 10.1037/pas0001000 33793263

[bibr22-10731911211033894] Chen F. F. (2008). What happens if we compare chopsticks with forks? The impact of making inappropriate comparisons in cross-cultural research. Journal of Personality and Social Psychology, 95(5), 1005-1018. 10.1037/a0013193 18954190

[bibr23-10731911211033894] Cheung M. W.-L. (2020). metaSEM (Version 1.2.5) [Computer software]. https://cran.r-project.org/web/packages/metaSEM/

[bibr24-10731911211033894] Cheung M. W.-L. (2013). Multivariate meta-analysis as structural equation models. Structural Equation Modeling: A Multidisciplinary Journal, 20(3), 429-454. 10.1080/10705511.2013.797827

[bibr25-10731911211033894] Cheung M. W.-L. Chan W. (2005a). Meta-analytic structural equation modeling: A two-stage approach. Psychological Methods, 10(1), 40-64. 10.1037/1082-989X.10.1.40 15810868

[bibr26-10731911211033894] Cheung M. W.-L. Chan W. (2005b). Classifying correlation matrices into relatively homogeneous subgroups: A cluster analytic approach. Educational and Psychological Measurement, 65(6), 954-979. 10.1177/0013164404273946

[bibr27-10731911211033894] Cheung M. W.-L. Chan W. (2009). A two-stage approach to synthesizing covariance matrices in meta-analytic structural equation modeling. Structural Equation Modeling: A Multidisciplinary Journal, 16(1), 28-53. 10.1080/10705510802561295

[bibr28-10731911211033894] Cheung M. W.-L. Cheung S. F. (2016). Random-effects models for meta-analytic structural equation modeling: Review, issues, and illustrations. Research Synthesis Methods, 7(2), 140-155. 10.1002/jrsm.1166 27286900

[bibr29-10731911211033894] *Chung U.-S. Rim H.‑D. Lee Y.‑H. Kim S.‑H. (2003). Comparison of reliability and validity of three Korean versions of the 20-item Toronto Alexithymia Scale. Korean Journal of Psychosomatic Medicine, 11(1), 77-88.

[bibr30-10731911211033894] *Cleland C. Magura S. Foote J. Rosenblum A. Kosanke N. (2005). Psychometric properties of the Toronto Alexithymia Scale (TAS-20) for substance users. Journal of Psychosomatic Research, 58(3), 299-306. 10.1016/j.jpsychores.2004.11.002 15865955

[bibr31-10731911211033894] Cochrane C. E. Brewerton T. D. Wilson D. B. Hodges E. L. (1993). Alexithymia in the eating disorders. International Journal of Eating Disorders, 14(2), 219-222. 10.1002/1098-108x(199309)14:2<219::aid-eat2260140212>3.0.co;2-g 8401555

[bibr32-10731911211033894] *Colombarolli M. S. Zuanazzi A. C. Miguel F. K. Giromini L. (2019). Psychometric properties of the Toronto Alexithymia Scale (TAS-20) in Brazil. Transcultural Psychiatry, 56(5), 992-1010. 10.1177/1363461519847312 31067154

[bibr33-10731911211033894] Corcos M. Guilbaud O. Speranza M. Paterniti S. Loas G. Stephan P. Jeammet P. (2000). Alexithymia and depression in eating disorders. Psychiatry Research, 93(3), 263-266. 10.1016/S0165-1781(00)00109-8 10760385

[bibr34-10731911211033894] Costa A. Peppe A. Carlesimo G. A. Salamone G. Caltagirone C. (2010). Prevalence and characteristics of alexithymia in Parkinson’s disease. Psychosomatics, 51(1), 22-28. 10.1016/S0033-3182(10)70655-1 20118437

[bibr35-10731911211033894] Cox B. J. Swinson R. P. Shulman I. D. Bourdeau D. (1995). Alexithymia in panic disorder and social phobia. Comprehensive Psychiatry, 36(3), 195-198. 10.1016/0010-440x(95)90081-6 7648842

[bibr36-10731911211033894] Craparo G. Faraci P. Gori A. (2015). Psychometric properties of the 20-item Toronto Alexithymia Scale in a group of Italian younger adolescents. Psychiatry Investigation, 12(4), 500-507. 10.4306/pi.2015.12.4.500 26508961 PMC4620307

[bibr37-10731911211033894] *de Gucht V. Fontaine J. Fischler B. (2004). Temporal stability and differential relationships with neuroticism and extraversion of the three subscales of the 20-item Toronto Alexithymia Scale in clinical and nonclinical samples. Journal of Psychosomatic Research, 57(1), 25-33. 10.1016/S0022-3999(03)00577-4 15256292

[bibr38-10731911211033894] DiStefano C. Motl R. W. (2006). Further investigating method effects associated with negatively worded items on self-report surveys. Structural Equation Modeling: A Multidisciplinary Journal, 13, 440-464. 10.1207/s15328007sem1303_6

[bibr39-10731911211033894] Eid M. Geiser C. Koch T. Heene M. (2017). Anomalous results in G-factor models: Explanations and alternatives. Psychological Methods, 22(3), 541-562. 10.1037/met0000083 27732052

[bibr40-10731911211033894] *Eiden T. C. (1998). Twenty item Toronto Alexithymia Scale: Construct validity in a college student population [Doctoral dissertation, Oklahoma State University]. Oklahoma State University Digital Archive. https://hdl.handle.net/11244/33514

[bibr41-10731911211033894] *El Abiddine F. Z. Dave H. Aldhafri S. El-Astal S. Hemaid F. Parker J. D. A. (2017). Cross-validation of the 20-item Toronto Alexithymia Scale: Results from an Arabic multicenter study. Personality and Individual Differences, 113(July), 219-222. 10.1016/j.paid.2017.03.017

[bibr42-10731911211033894] *Erni T. Lötscher K. Modestin J. (1997). Two-factor solution of the 20-ltem Toronto Alexithymia Scale confirmed. Psychopathology, 30(6), 335-340. 10.1159/000285079 9444703

[bibr43-10731911211033894] *Fournier A. Luminet O. Dambrun M. Dutheil F. Pellissier S. Mondillon L. (2019). Importance of considering interoceptive abilities in alexithymia assessment. PeerJ, 7, Article e7615. 10.7717/peerj.7615 PMC6874858 31768300

[bibr44-10731911211033894] Franz M. Popp K. Schaefer R. Sitte W. Schneider C. Hardt J. Decker O. Braehler E. (2008). Alexithymia in the German general population. Social Psychiatry and Psychiatric Epidemiology, 43(1), 54-62. 10.1007/s00127-007-0265-1 17934682

[bibr45-10731911211033894] *Franz M. Schneider C. Schäfer R. Schmitz N. Zweyer K. (2001). Faktorenstruktur und Testgütekriterien der deutschen Version der Toronto-Alexithymie-Skala (TAS-20) bei psychosomatischen Patienten [Factorial structure and psychometric properties of the German version of the Toronto Alexithymia Scale (TAS-20) of psychosomatic patients]. PPmP—Psychotherapie Psychosomatik Medizinische Psychologie, 51(2), 48-55. 10.1055/s-2001-10755 11268779

[bibr46-10731911211033894] *Fukunishi I. Nakagawa T. Nakamura H. Kikuchi M. Takubo M. (1997). Is alexithymia a culture-bound construct? Validity and reliability of the Japanese versions of the 20-item Toronto Alexithymia Scale and modified Beth Israel Hospital Psychosomatic Questionnaire. Psychological Reports, 80(3), 787-799. 10.2466/pr0.1997.80.3.787 9198380

[bibr47-10731911211033894] Gaskin C. J. Lambert S. D. Bowe S. J. Orellana L. (2017). Why sample selection matters in exploratory factor analysis: Implications for the 12-item World Health Organization Disability Assessment Schedule 2.0. BMC Medical Research Methodology, 17(1), 1-9. 10.1186/s12874-017-0309-5 28283019 PMC5346210

[bibr48-10731911211033894] Geisinger K. F. McCormick C. (2012). Testing and assessment in cross-cultural psychology. In Weiner I. (Ed.), Handbook of psychology (pp. 114-139). John Wiley. 10.1002/9781118133880.hop210005

[bibr49-10731911211033894] *Gignac G. E. Palmer B. R. Stough C. (2007). A confirmatory factor analytic investigation of the TAS–20: Corroboration of a five-factor model and suggestions for improvement. Journal of Personality Assessment, 89(3), 247-257. 10.1080/00223890701629730 18001225

[bibr50-10731911211033894] Gnambs T. Scharl A. Schroeders U. (2018). The structure of the Rosenberg Self-Esteem Scale: A cross-cultural meta-analysis. Zeitschrift Für Psychologie, 226(1), 14-29. 10.1027/2151-2604/a000317

[bibr51-10731911211033894] Gnambs T. Schroeders U. (2020). Cognitive abilities explain wording effects in the Rosenberg Self-Esteem Scale. Assessment, 27(2), 404-418. 10.1177/1073191117746503 29254352

[bibr52-10731911211033894] Gnambs T. Staufenbiel T. (2016). Parameter accuracy in meta-analyses of factor structures. Research Synthesis Methods, 7(2), 168-186. 10.1002/jrsm.1190 27286902

[bibr53-10731911211033894] Gnambs T. Staufenbiel T. (2018). The structure of the General Health Questionnaire (GHQ-12): Two meta-analytic factor analyses. Health Psychology Review, 12(2), 179-194. 10.1080/17437199.2018.1426484 29325498

[bibr54-10731911211033894] Goldberg L. R. (2006). Doing it all bass-ackwards: The development of hierarchical factor structures from the top down. Journal of Research in Personality, 40(4), 347-358. 10.1016/j.jrp.2006.01.001

[bibr55-10731911211033894] González-Arias M. Martínez-Molina A. Galdames S. Urzúa A. (2018). Psychometric properties of the 20-item Toronto Alexithymia Scale in the Chilean population. Frontiers in Psychology, 9, Article 963. 10.3389/fpsyg.2018.00963 PMC6005868 29946289

[bibr56-10731911211033894] *Güleç H. Köse S. Güleç M. Y. Çitak S. Evren C. Borckardt J. Sayar K. (2009). Reliability and factorial validity of the Turkish version of the 20-item Toronto Alexithymia Scale (TAS-20). Bulletin of Clinical Psychopharmacology, 19(3), 214-220.

[bibr57-10731911211033894] Haviland M. G. Reise S. P. (1996). Structure of the twenty-item Toronto Alexithymia Scale. Journal of Personality Assessment, 66(1), 116-125. 10.1207/s15327752jpa6601_9 8576826

[bibr58-10731911211033894] Heene M. Hilbert S. Draxler C. Ziegler M. Bühner M. (2011). Masking misfit in confirmatory factor analysis by increasing unique variances: A cautionary note on the usefulness of cutoff values of fit indices. Psychological Methods, 16(3), 319-336. 10.1037/a0024917 21843002

[bibr59-10731911211033894] Hirschfeld G. von Brachel R. Thielsch M. (2014). Selecting items for Big Five questionnaires: At what sample size do factor loadings stabilize? Journal of Research in Personality, 53(December), 54-63. 10.1016/j.jrp.2014.08.003

[bibr60-10731911211033894] Holzinger K. Swineford F. (1939). A study in factor analysis: The stability of a bifactor solution. University of Chicago Press.

[bibr61-10731911211033894] Honkalampi K. Hintikka J. Tanskanen A. Lehtonen J. Viinamäki H. (2000). Depression is strongly associated with alexithymia in the general population. Journal of Psychosomatic Research, 48(1), 99-104. 10.1016/S0022-3999(99)00083-5 10750635

[bibr62-10731911211033894] Horn J. L. (1965). A rationale and test for the number of factors in factor analysis. Psychometrika, 30(2), 179-185. 10.1007/BF02289447 14306381

[bibr63-10731911211033894] Iglesias-Rey M. Barreiro-de Acosta M. Caamaño-Isorna F. Vázquez Rodríguez I. Lorenzo González A. Bello-Paderne X. Domínguez-Muñoz J. E. (2012). Influence of alexithymia on health-related quality of life in inflammatory bowel disease: Are there any related factors? Scandinavian Journal of Gastroenterology, 47(4), 445-453. 10.3109/00365521.2012.654403 22300356

[bibr64-10731911211033894] Jankowsky K. Olaru G. Schroeders U. (2020). Compiling measurement invariant short scales in cross-cultural personality assessment using ant colony optimization. European Journal of Personality, 34(3), 470-485. 10.1002/per.2260

[bibr65-10731911211033894] Jak S. (2015). Meta-analytic structural equation modelling. Springer. 10.1007/978-3-319-27174-3

[bibr66-10731911211033894] Jak S. Cheung M. W.-L. (2018). Testing moderator hypotheses in meta-analytic structural equation modeling using subgroup analysis. Behavior Research Methods, 50(4), 1359-1373. 10.3758/s13428-018-1046-3 29869223 PMC6096661

[bibr67-10731911211033894] *Joukamaa M. Miettunen J. Kokkonen P. Koskinen M. Julkunen J. Kauhanen J. Jokelainen J. Veijola J. Läksy K. Järvelin M. R. (2001). Psychometric properties of the Finnish 20-item Toronto Alexithymia Scale. Nordic Journal of Psychiatry, 55(2), 123-127. 10.1080/08039480116694 11802910

[bibr68-10731911211033894] *Kamm J. S. G. (2016). High alexithymia in Chilean indigenous and Hispanic adolescent population: A cross cultural study [Doctoral dissertation, Justus-Liebig-Universität Gießen]. Giessener Elektronische Bibliothek. http://geb.uni-giessen.de/geb/volltexte/2017/13373/

[bibr69-10731911211033894] *Khosravani V. Najafi M. Naragon-Gainey K. Mohammadzadeh A. (2019). Investigation of the factorial structure and psychometric properties of the Persian version of the Toronto Alexithymia Scale-20 in patients with psychiatric disorders. Current Psychology. Advance online publication. 10.1007/s12144-019-00329-3

[bibr70-10731911211033894] Kirmayer L. J. (1987). Languages of suffering healing: Alexithymia as a social and cultural process. Transcultural Psychiatric Research Review, 24(2), 119-136. 10.1177/136346158702400204

[bibr71-10731911211033894] *Koch A. S. Kleiman A. Wegener I. Zur B. Imbierowicz K. Geiser F. Conrad R. (2015). Factorial structure of the 20-item Toronto Alexithymia Scale in a large sample of somatoform patients. Psychiatry Research, 225(3), 355-363. 10.1016/j.psychres.2014.12.013 25613660

[bibr72-10731911211033894] *Kojima M. Frasure-Smith N. Lespérance F. (2001). Alexithymia following myocardial infarction: Psychometric properties and correlates of the Toronto Alexithymia Scale. Journal of Psychosomatic Research, 51(3), 487-495. 10.1016/S0022-3999(01)00253-7 11602218

[bibr73-10731911211033894] *Komaki G. Maeda M. Arimura T. Nakata A. Shinoda H. Ogata I. Shimura M. Kawamura N. Kubo C. (2003). The reliability and factorial validity of the Japanese version of the 20-item Toronto Alexithymia Scale (TAS-20). Japanese Journal of Psychosomatic Research, 43, 839-846. 10.1016/S0022-3999(03)00360-X

[bibr74-10731911211033894] Kooiman C. G. Spinhoven P. Trijsburg R. W. (2002). The assessment of alexithymia: A critical review of the literature and a psychometric study of the Toronto Alexithymia Scale-20. Journal of Psychosomatic Research, 53(6), 1083-1090. 10.1016/s0022-3999(02)00348-3 12479990

[bibr75-10731911211033894] Krippendorff K. (2013). Content analysis: An introduction to its methodology. Sage.

[bibr76-10731911211033894] *Kroner D. G. Forth A. E. (1995). The Toronto Alexithymia Scale with incarcerated offenders. Personality and Individual Differences, 19(5), 625-634. 10.1016/0191-8869(95)00116-N

[bibr77-10731911211033894] *Larwood J. L. Vanman E. J. Dingle G. A. (2020). Negative valence specific deficits in judgements of musical affective quality in alexithymia. Cognition and Emotion, 35(3), 500-509. 10.1080/02699931.2019.1707514 31906793

[bibr78-10731911211033894] *La-Salete Rocha Nunes J. (2011). Estudo das qualidades psicométricas da Escala de Alexitimia de Toronto (20 itens) numa amostra portuguesa de adolescentes [Study of the psychometric qualities of the Toronto Alexithymia Scale (20 items) in a Portuguese sample of adolescents] [Doctoal dissertation, Universidade do Porto]. Repositório Aberto. https://core.ac.uk/download/pdf/162558983.pdf

[bibr79-10731911211033894] Leising D. Grande T. Faber R. (2009). The Toronto Alexithymia Scale (TAS-20): A measure of general psychological distress. Journal of Research in Personality, 43(4), 707-710. 10.1016/j.jrp.2009.03.009

[bibr80-10731911211033894] Leweke F. Leichsenring F. Kruse J. Hermes S. (2012). Is alexithymia associated with specific mental disorders? Psychopathology, 45(1), 22-28. 10.1159/000325170 22123513

[bibr81-10731911211033894] Lewin K. (1952). Field theory in social science: Selected theoretical papers by Kurt Lewin. Tavistock.

[bibr82-10731911211033894] *Loas G. Corcos M. Stephan P. Pellet J. Bizouard P. Venisse J. L. Perez-Diaz F. Guelfi J. D. Jeammet P. (2001). Factorial structure of the 20-item Toronto Alexithymia Scale: Confirmatory factorial analyses in nonclinical and clinical samples. Journal of Psychosomatic Research, 50(5), 255-261. 10.1016/S0022-3999(01)00197-0 11399282

[bibr83-10731911211033894] Loas G. Otmani O. Verrier A. Fremaux D. Marchand M. P. (1996). Factor analysis of the French version of the 20-item Toronto Alexithymia Scale (TAS-20). Psychopathology, 29(2), 139-144. 10.1159/000284983 8861519

[bibr84-10731911211033894] *Loiselle C. G. Cossette S. (2001). Cross-cultural validation of the Toronto Alexithymia Scale (TAS-20) in U.S. and Peruvian populations. Transcultural Psychiatry, 38(3), 348-362. 10.1177/136346150103800305

[bibr85-10731911211033894] Lorenzo-Seva U. ten Berge J. M. F. (2006). Tucker’s congruence coefficient as a meaningful index of factor similarity. Methodology: European Journal of Research Methods for the Behavioral and Social Sciences, 2(2), 57-64. 10.1027/1614-2241.2.2.57

[bibr86-10731911211033894] *Louth S. M. (1998). Alexithymia and the capacity to evaluate states of affect and pain [Doctoral dissertation, University of British Columbia]. UBC Library Open Collections. https://open.library.ubc.ca/collections/831/items/1.0088755

[bibr87-10731911211033894] MacCallum R. C. Widaman K. F. Preacher K. J. Hong S. (2001). Sample size in factor analysis: The role of model error. Multivariate Behavioral Research, 36(4), 611-637. 10.1207/S15327906MBR3604_06 26822184

[bibr88-10731911211033894] Maggini C. Raballo A. (2004). Alexithymia and schizophrenic psychopathology. Acta Bio-Medica: Atenei Parmensis, 75(1), 40-49. 15315086

[bibr89-10731911211033894] Marsh H. W. Grayson D. (1995). Latent variable models of multitrait-multimethod data. In Hoyle R. H. (Ed.), Structural equation modeling: Concepts, issues, and applications (pp. 177-198). Sage.

[bibr90-10731911211033894] *Martínez Sánchez F. (1996). Adaptación española de la escala de Alexitimia de Toronto (TAS-20) [The Spanish version of the Toronto Alexithymia Scale (TAS-20)]. Clínica y Salud, 7(1), 19-32.

[bibr91-10731911211033894] Marty P. M'Uzan M. (1963). La pensée opératoire [The Operational thought]. Revue Française de Psychanalyse, 27, 345-356.

[bibr92-10731911211033894] Matsumoto D. Yoo S. H. Fontaine J. (2008). Mapping expressive differences around the world: The relationship between emotional display rules and individualism versus collectivism. Journal of Cross-Cultural Psychology, 39(1), 55-74. 10.1177/0022022107311854

[bibr93-10731911211033894] Mattila A. K. Kronholm E. Jula A. Salminen J. K. Koivisto A. M. Mielonen R. L. Joukamaa M. (2008). Alexithymia and somatization in general population. Psychosomatic Medicine, 70(6), 716-722. 10.1097/PSY.0b013e31816ffc39 18596251

[bibr94-10731911211033894] Mayer J. D. Salovey P. Caruso D. R. Sitarenios G. (2003). Measuring emotional intelligence with the MSCEIT V2.0. Emotion, 3(1), 97-105. 10.1037/1528-3542.3.1.97 12899321

[bibr95-10731911211033894] McDonald R. P. (1999). Test theory: A unified treatment. Lawrence Erlbaum.

[bibr96-10731911211033894] *Meganck R. Vanheule S. Desmet M. (2008). Factorial validity and measurement invariance of the 20-Item Toronto Alexithymia Scale in clinical and nonclinical samples. Assessment, 15(1), 36-47. 10.1177/1073191107306140 18258730

[bibr97-10731911211033894] *Moriguchi Y. Maeda M. Igarashi T. Ishikawa T. Shoji M. Kubo C. Komaki G. (2007). Age and gender effect on alexithymia in large, Japanese community and clinical samples: A cross-validation study of the Toronto Alexithymia Scale (TAS-20). BioPsychoSocial Medicine, 1(1), Article 7. 10.1186/1751-0759-1-7 PMC1838425 17371586

[bibr98-10731911211033894] Mulaik S. A. Quartetti D. A. (1997). First order or higher order general factor? Structural Equation Modeling: A Multidisciplinary Journal, 4(3), 193-211. 10.1080/10705519709540071

[bibr99-10731911211033894] *Müller J. Bühner M. Ellgring H. (2003). Is there a reliable factorial structure in the 20-item Toronto Alexithymia Scale? A comparison of factor models in clinical and normal adult samples. Journal of Psychosomatic Research, 55(6), 561-568. 10.1016/S0022-3999(03)00033-3 14642988

[bibr100-10731911211033894] Neale M. C. Hunter M. D. Pritikin J. N. Zahery M. Brick T. R. Kirkpatrick R. M. Estabrook R. Bates T. C. Maes H. H. Boker S. M. (2016). OpenMx 2.0: Extended structural equation and statistical modeling. Psychometrika, 81(2), 535-549. 10.1007/s11336-014-9435-8 25622929 PMC4516707

[bibr101-10731911211033894] Nemiah J. C. Sifneos P. E. (1970). Psychosomatic illness: A problem in communication. Psychotherapy and Psychosomatics, 18(1-6), 154-160. 10.1159/000286074 5520658

[bibr102-10731911211033894] *Pandey R. Mandal M. K. Taylor G. J. Parker J. D. A. (1996). Cross-cultural alexithymia: Development and validation of a Hindi translation of the 20-item Toronto Alexithymia Scale. Journal of Clinical Psychology, 52(2), 173-176. https://doi.org/10.1002/(SICI)1097-4679(199603)52:2<173::AID-JCLP8>3.0.CO;2-V 8771445 10.1002/(SICI)1097-4679(199603)52:2<173::AID-JCLP8>3.0.CO;2-V

[bibr103-10731911211033894] *Parker J. D. A. Bagby R. M. Taylor G. J. Endler N. S. Schmitz P. (1993). Factorial validity of the 20-item Toronto Alexithymia Scale. European Journal of Personality, 7(4), 221-232. 10.1002/per.2410070403

[bibr104-10731911211033894] *Parker J. D. A. Eastabrook J. M. Keefer K. V. Wood L. M. (2010). Can alexithymia be assessed in adolescents? Psychometric properties of the 20-item Toronto Alexithymia Scale in younger, middle, and older adolescents. Psychological Assessment, 22(4), 798-808. 10.1037/a0020256 20804260

[bibr105-10731911211033894] *Parker J. D. A. Shaughnessy P. A. Wood L. M. Majeski S. A. Eastabrook J. M. (2005). Cross-cultural alexithymia: Validity of the 20-item Toronto Alexithymia Scale in North American aboriginal populations. Journal of Psychosomatic Research, 58(1), 83-88. 10.1016/j.jpsychores.2004.06.003 15771874

[bibr106-10731911211033894] *Parker J. D. A. Taylor G. J. Bagby R. M. (2003). The 20-item Toronto Alexithymia Scale: III. Reliability and factorial validity in a community population. Journal of Psychosomatic Research, 55(3), 269-275. 10.1016/S0022-3999(02)00578-0 12932802

[bibr107-10731911211033894] *Pinaquy S. Chabrol H. Barbe P. (2002). Factorial analysis and internal consistency of the French version of the Toronto Alexithymia Scale (TAS 20), in obese women. L'Encephale, 28(4), 277-282. https://europepmc.org/article/med/12232536 12232536

[bibr108-10731911211033894] *Popp K. Schäfer R. Schneider C. Brähler E. Decker O. Hardt J. Franz M. (2008). Faktorstruktur und Reliabilität der Toronto-Alexithymie-Skala (TAS-20) in der deutschen Bevölkerung [Factor structure and reliability of the Toronto Alexithymia Scale (TAS−20) in the German Population]. PPmP—Psychotherapie Psychosomatik Medizinische Psychologie, 58(5), 208-214. 10.1055/s-2007-986196 17893837

[bibr109-10731911211033894] *Praceres N. Parker J. D. A. Taylor G. J. (2012). Adaptação Portuguesa da Escala de Alexitimia de Toronto de 20 Itens (TAS-20) [Portuguese adaptation of the 20-item Toronto Alexithymia Scale (TAS-20)]. Revista Iberoamericana de Diagnostico y Evaluacion Psicologica, 9, 9-21.

[bibr110-10731911211033894] *Preece D. A. Becerra R. Allan A. Robinson K. Chen W. Hasking P. Gross J. J. (2020). Assessing alexithymia: Psychometric properties of the Perth Alexithymia Questionnaire and 20-item Toronto Alexithymia Scale in United States adults. Personality and Individual Differences, 166, Article 110138. 10.1016/j.paid.2020.110138

[bibr111-10731911211033894] Preece D. A. Becerra R. Robinson K. Allan A. Boyes M. Chen W. Hasking P. Gross J. J. (2020). What is alexithymia? Using factor analysis to establish its latent structure and relationship with fantasizing and emotional reactivity. Journal of Personality, 88(6), 1162-1176. 10.1111/jopy.12563 32463926

[bibr112-10731911211033894] *Preece D. A. Becerra R. Robinson K. Dandy J. (2018). Assessing alexithymia: Psychometric properties and factorial invariance of the 20-item Toronto Alexithymia Scale in nonclinical and psychiatric samples. Journal of Psychopathology and Behavioral Assessment, 40(2), 276-287. 10.1007/s10862-017-9634-6

[bibr113-10731911211033894] Preece D. A. Becerra R. Robinson K. Dandy J. Allan A. (2018). The psychometric assessment of alexithymia: Development and validation of the Perth Alexithymia Questionnaire. Personality and Individual Differences, 132(October), 32-44. 10.1016/j.paid.2018.05.011

[bibr114-10731911211033894] *Preece D. A. Parry C. L. Allan M. M. Allan A. (2021). Assessing alexithymia in forensic settings: Psychometric properties of the 20-item Toronto Alexithymia Scale among incarcerated adult offenders. Criminal Behaviour and Mental Health, 31(1), 31-43. 10.1002/cbm.2176 33200532

[bibr115-10731911211033894] Reise S. P. (2012). The rediscovery of bifactor measurement models. Multivariate Behavioral Research, 47(5), 667-696. 10.1080/00273171.2012.715555 24049214 PMC3773879

[bibr116-10731911211033894] *Reise S. P. Bonifay W. E. Haviland M. G. (2013). Scoring and modeling psychological measures in the presence of multidimensionality. Journal of Personality Assessment, 95(2), 129-140. 10.1080/00223891.2012.725437 23030794

[bibr117-10731911211033894] Reise S. P. Moore T. M. Haviland M. G. (2010). Bifactor models and rotations: Exploring the extent to which multidimensional data yield univocal scale scores. Journal of Personality Assessment, 92(6), 544-559. 10.1080/00223891.2010.496477 20954056 PMC2981404

[bibr118-10731911211033894] Revelle W. (2020). psych (Version 2.0.12) [Computer software]. https://cran.r-project.org/web/packages/psych/

[bibr119-10731911211033894] *Richards H. L. Fortune D. G. Griffiths C. E. Main C. J. (2005). Alexithymia in patients with psoriasis: Clinical correlates and psychometric properties of the Toronto Alexithymia Scale-20. Journal of Psychosomatic Research, 58(1), 89-96. 10.1016/j.jpsychores.2004.03.009 15771875

[bibr120-10731911211033894] Ruscio J. Roche B. (2012). Determining the number of factors to retain in an exploratory factor analysis using comparison data of known factorial structure. Psychological Assessment, 24(2), 282-292. 10.1037/a0025697 21966933

[bibr121-10731911211033894] Ryder A. G. Sunohara M. Dere J. Chentsova-Dutton Y. E. (2018). The cultural shaping of alexithymia. In Luminet O. Bagby R. M. Taylor G. J. (Eds.), Alexithymia: Advances in research, theory, and clinical practice (pp. 33-48), Cambridge University Press.

[bibr122-10731911211033894] Schermelleh-Engel K. Moosbrugger H. Müller H. (2003). Evaluating the fit of structural equation models: Tests of significance and descriptive goodness-of-fit measures. Methods of Psychological Research, 8(2), 23-74.

[bibr123-10731911211033894] Schmid J. Leiman J. M. (1957). The development of hierarchical factor solutions. Psychometrika, 22(1), 53-61. 10.1007/BF02289209

[bibr124-10731911211033894] Schroeders U. Gnambs T. (2020). Degrees of freedom in multigroup confirmatory factor analyses: Are models of measurement invariance testing correctly specified? European Journal of Psychological Assessment, 36(1), 105-113. 10.1027/1015-5759/a000500

[bibr125-10731911211033894] Schroeders U. Wilhelm O. Olaru G. (2016). Meta-heuristics in short scale construction: Ant colony optimization and genetic algorithm. PLOS ONE, 11, Article e0167110. 10.1371/journal.pone.0167110 PMC5125670 27893845

[bibr126-10731911211033894] Sekely A. Bagby R. M. Porcelli P. (2018). Assessment of the alexithymia construct. In Luminet O. Bagby R. M. Taylor G. J. (Eds.), Alexithymia: Advances in research, theory, and clinical practice (pp. 17-32). Cambridge University Press.

[bibr127-10731911211033894] *Seo S. S. Chung U.-S. Rim H. D. Jeong S. H. (2009). Reliability and validity of the 20-item Toronto Alexithymia Scale in Korean adolescents. Psychiatry Investigation, 6(3), 173-179. 10.4306/pi.2009.6.3.173 20046392 PMC2796065

[bibr128-10731911211033894] Sifneos P. E. (1973). The prevalence of “alexithymic” characteristics in psychosomatic patients. Psychotherapy and Psychosomatics, 22, 255-262. 10.1159/000286529 4770536

[bibr129-10731911211033894] *Simonsson-Sarnecki M. Lundh L.‑G. Törestad B. Bagby R. M. Taylor G. J. Parker J. D. A. (2000). A Swedish translation of the 20-item Toronto Alexithymia Scale: Cross-validation of the factor structure. Scandinavian Journal of Psychology, 41(1), 25-30. 10.1111/1467-9450.00167 10731840

[bibr130-10731911211033894] Spitzer C. Siebel-Jürges U. Barnow S. Grabe H. J. Freyberger H. J. (2005). Alexithymia and interpersonal problems. Psychotherapy and Psychosomatics, 74(4), 240-246. 10.1159/000085148 15947514

[bibr131-10731911211033894] Taylor G. J. Bagby R. M. (2020). Examining proposed changes to the conceptualization of the alexithymia construct: The way forward tilts to the past. Psychotherapy and Psychosomatics, 90, 145-155. 10.1159/000511988 33285546

[bibr132-10731911211033894] Taylor G. J. Bagby R. M. Parker J. D. A. (2003). The 20-item Toronto Alexithymia Scale: IV. reliability and factorial validity in different languages and cultures. Journal of Psychosomatic Research, 55(3), 277-283. 10.1016/S0022-3999(02)00601-3 12932803

[bibr133-10731911211033894] Taylor G. J. Bagby R. M. Parker J. D. A. (1992). The revised Toronto Alexithymia Scale: Some reliability, validity, and normative data. Psychotherapy and Psychosomatics, 57(1-2), 34–41. 10.1159/000288571 1584897

[bibr134-10731911211033894] Taylor G. J. Parker J. D. Bagby R. M. (1990). A preliminary investigation of alexithymia in men with psychoactive substance dependence. American Journal of Psychiatry, 147(9), 1228-1230. https://doi.org10.1176/ajp.147.9.1228 2386256 10.1176/ajp.147.9.1228

[bibr135-10731911211033894] Taylor G. J. Ryan D. Bagby R. M. (1985). Toward the development of a new self-report alexithymia scale. Psychotherapy and Psychosomatics, 44(4), 191-199. 10.1159/000287912 3837277

[bibr136-10731911211033894] *Thorberg F. A. Young R. M. Sullivan K. A. Lyvers M. Hurst C. Connor J. P. Feeney G. F. (2010). A confirmatory factor analysis of the Toronto Alexithymia Scale (TAS-20) in an alcohol-dependent sample. Psychiatry Research, 178(3), 565-567. 10.1016/j.psychres.2009.09.015 20510467

[bibr137-10731911211033894] *Tuliao A. P. Klanecky A. K. Landoy B. V. N. McChargue D. E. (2020). Toronto Alexithymia Scale–20: Examining 18 competing factor structure solutions in a U.S. sample and a Philippines sample. Assessment, 27(7), 1515-1531. 10.1177/1073191118824030 30661362

[bibr138-10731911211033894] van de Vijver F. J. R. He J. (2016). Bias assessment and prevention in noncognitive outcome measures in context assessments. In Kuger S. Klieme E. Jude N. Kaplan D. (Eds.), Assessing contexts of learning (pp. 229-253). Springer. 10.1007/978-3-319-45357-6_9

[bibr139-10731911211033894] van de Vijver F. J. R. Tanzer N. K. (2004). Bias and equivalence in cross-cultural assessment: An overview. European Review of Applied Psychology, 54(2), 119-135. 10.1016/j.erap.2003.12.004

[bibr140-10731911211033894] van Erp S. Verhagen J. Grasman R. P. P. P. Wagenmakers E.-J. (2017). Estimates of between-study heterogeneity for 705 meta-analyses reported in Psychological Bulletin from 1990–2013. Journal of Open Psychology Data, 5(1), 4. 10.5334/jopd.33

[bibr141-10731911211033894] Velicer W. F. (1976). Determining the number of components from the matrix of partial correlations. Psychometrika, 41(3), 321-327. 10.1007/BF02293557

[bibr142-10731911211033894] *Verissimo R. (2001). The Portuguese version of the 20-item Toronto Alexithymia Scale: I. Linguistic adaptation, semantic validation, and reliability study. Acta Médica Portuguesa, 14(5-6), 529-536. https://www.actamedicaportuguesa.com/revista/index.php/amp/article/view/1884 11878167

[bibr143-10731911211033894] Vorst H. C. Bermond B. (2001). Validity and reliability of the Bermond-Vorst Alexithymia Questionnaire. Personality and Individual Differences, 30(3), 413-434. 10.1016/S0191-8869(00)00033-7

[bibr144-10731911211033894] *Watters C. A. Taylor G. J. Ayearst L. E. Bagby R. M. (2016). Measurement invariance of English and French language versions of the 20-Item Toronto Alexithymia Scale. European Journal of Psychological Assessment, 35(1), 29-36. 10.1027/1015-5759/a000365

[bibr145-10731911211033894] Williams Z. J. Gotham K. O. (2021). Improving the measurement of alexithymia in autistic adults: A psychometric investigation and refinement of the twenty-item Toronto Alexithymia Scale. Molecular Autism, 12(1), Article 20. 10.1186/s13229-021-00427-9 PMC7971146 33653400

[bibr146-10731911211033894] *Wise T. N. Simpson N. Sheridan M. J. (2000). Comparison of 26-item and 20-item versions of the Toronto Alexithymia Scale for psychiatric outpatients. Psychological Reports, 87(1), 127-132. 10.2466/pr0.2000.87.1.127 11026400

[bibr147-10731911211033894] Wright A. G. C. Thomas K. M. Hopwood C. J. Markon K. E. Pincus A. L. Krueger R. F. (2012). The hierarchical structure of *DSM-5* pathological personality traits. Journal of Abnormal Psychology, 121(4), 951-957. 10.1037/a0027669 22448740 PMC3389150

[bibr148-10731911211033894] Yun S. Shin J. Lee T. (2019). A study of factor structure of the Korean version of the 20-item Toronto Alexithymia Scale. Stress, 27(4), 380-388. 10.17547/kjsr.2019.27.4.380

[bibr149-10731911211033894] *Zhu X. Yi J. Yao S. Ryder A. G. Taylor G. J. Bagby R. M. (2007). Cross-cultural validation of a Chinese translation of the 20-item Toronto Alexithymia Scale. Comprehensive Psychiatry, 48(5), 489-496. 10.1016/j.comppsych.2007.04.007 17707259

[bibr150-10731911211033894] *Zimmermann G. Rossier J. Meyer de Stadelhofen F. Gaillard F. (2005). Alexithymia assessment and relations with dimensions of personality. European Journal of Psychological Assessment, 21(1), 23-33. 10.1027/1015-5759.21.1.23

