# Supplementary material for: The Structure of the Toronto Alexithymia Scale (TAS-20): A Meta-Analytic Confirmatory Factor Analysis
Source: Assessment. 2021 Jul 26;29(8):1806–23. doi: 10.1177/10731911211033894 (PMC9597132; doi:10.1177/10731911211033894)

Content

Table OS 1. Parameter Estimates of Competing Measurement Models for the TAS-20 2
Table OS 2. Factor Model Fit of the TAS-20 for The English Version 7
Table OS 3. Parameter Estimates of the TAS-20 Across Psychiatric Status 8
Figure OS 1. Correlation Matrix of All Correlation Matrices 9

Table OS 1

Parameter Estimates of Competing Measurement Models for the TAS-20

	Model 1		Model 2			Model 3a		
Factor(s)	Alex		DIF/DDF	EOT		DIF	DDF	EOT
λ_1	.69	λ_1	.69		λ_1	.69		
λ_2	.72	λ_2	.71		λ_2		.74	
λ_3	.51	λ_3	.51		λ_3	.52		
λ_4	.55	λ_4	.54		λ_4		.57	
λ_5	.19	λ_5		.35	λ_5			.35
λ_6	.64	λ_6	.65		λ_6	.65		
λ_7	.60	λ_7	.60		λ_7	.61		
λ_8	.30	λ_8		.38	λ_8			.37
λ_9	.73	λ_9	.73		λ_9	.74		
λ_{10}	.33	λ_{10}		.51	λ_{10}			.52
λ_{11}	.64	λ_{11}	.62		λ_{11}		.64	
λ_{12}	.51	λ_{12}	.51		λ_{12}		.52	
λ_{13}	.74	λ_{13}	.74		λ_{13}	.75		
λ_{14}	.63	λ_{14}	.63		λ_{14}	.63		
λ_{15}	.35	λ_{15}		.43	λ_{15}			.43
λ_{16}	.23	λ_{16}		.31	λ_{16}			.31
λ_{17}	.54	λ_{17}	.52		λ_{17}		.55	
λ_{18}	.22	λ_{18}		.39	λ_{18}			.39
λ_{19}	.34	λ_{19}		.55	λ_{19}			.57
λ_{20}	.26	λ_{20}		.33	λ_{20}			.32
		$r_{\text{DIF/DDF, EO}}$	.43		$r_{\text{DIF, DDF}}$	.77		
					$r_{\text{DDF, EOT}}$	.47		
					$r_{\text{DIF, EOT}}$	.32		
Model fit								
χ^2	29,912.4	χ^2	16,757.7		χ^2	8,424.2		
df	170	*df*	169		*df*	167		
CFI	.768	CFI	.871		CFI	.936		
RMSEA	.050	RMSEA	.038		RMSEA	.027		
SRMR	.084	SRMR	.056		SRMR	.041		

	Model 3b				Model 3c		
Factor(s)	DIF/DDF	PT	IOE		DIF/DDF	EOT	IOE
λ_1	.69			λ_1	.68		
λ_2	.71			λ_2	.70		
λ_3	.51			λ_3	.51		
λ_4	.54			λ_4			.65
λ_5		.35		λ_5			.32
λ_6	.65			λ_6	.64		
λ_7	.60			λ_7	.60		
λ_8		.39		λ_8		.36	
λ_9	.73			λ_9	.73		
λ_{10}			.52	λ_{10}			.48
λ_{11}	.62			λ_{11}	.62		
λ_{12}	.51			λ_{12}	.50		
λ_{13}	.74			λ_{13}	.74		
λ_{14}	.63			λ_{14}	.63		
λ_{15}			.43	λ_{15}		.42	
λ_{16}			.32	λ_{16}		.30	
λ_{17}	.52			λ_{17}		.59	
λ_{18}			.39	λ_{18}			.37
λ_{19}			.56	λ_{19}			.52
λ_{20}		.34		λ_{20}		.32	
$r_{\text{DIF/DDF, PT}}$	.46			$r_{\text{DIF/DDF, IOE}}$	.61		
$r_{\text{DIF/DDF, IOE}}$	.41			$r_{\text{DIF/DDF, EOT}}$	.81		
$r_{\text{PT, IOE}}$	.94			$r_{\text{IOE, EOT}}$	.61		
Model fit							
χ^2	16,635.4			χ^2	22,216.9		
df	167			*df*	167		
CFI	.872			CFI	.828		
RMSEA	.038			RMSEA	.044		
SRMR	.055			SRMR	.073		

	Model 4					Model 5			
Factor(s)	DIF	DDF	PT	IOE		Alex	DIF#	DDF#	EOT#
λ_1	.69				λ_1	.56	.41		
λ_2		.74			λ_2	.74		.07	
λ_3	.52				λ_3	.38	.37		
λ_4		.57			λ_4	.54		.35	
λ_5			.36		λ_5	.09			.37
λ_6	.65				λ_6	.51	.41		
λ_7	.61				λ_7	.45	.44		
λ_8			.38		λ_8	.25			.25
λ_9	.74				λ_9	.59	.45		
λ_{10}				.53	λ_{10}	.17			.51
λ_{11}		.65			λ_{11}	.66		.00	
λ_{12}		.52			λ_{12}	.53		.02	
λ_{13}	.75				λ_{13}	.60	.45		
λ_{14}	.63				λ_{14}	.50	.40		
λ_{15}				.43	λ_{15}	.31			.26
λ_{16}				.32	λ_{16}	.20			.21
λ_{17}		.55			λ_{17}	.56		.05	
λ_{18}				.40	λ_{18}	.10			.41
λ_{19}				.57	λ_{19}	.17			.58
λ_{20}			.33		λ_{20}	.21			.22
$r_{\text{DIF, DDF}}$	.77		$r_{\text{PT, IOE}}$	.94					
$r_{\text{DDF, IOE}}$	.46		$r_{\text{DDF, PT}}$	.47					
$r_{\text{DIF, IOE}}$	.30		$r_{\text{DIF, PT}}$	.36					
Model fit									
χ^2	8,274.7				χ^2	6,159.8			
df	164				*df*	150			
CFI	.937				CFI	.953			
RMSEA	.027				RMSEA	.024			
SRMR	.040				SRMR	.026			

	Model 5b				Model 6			
Factor(s)	Alex	DIF[#]	EOT[#]		DIF	DDF	EOT	Method
λ_1	.55	.42		λ_1	.69			
λ_2	.75			λ_2		.75		
λ_3	.38	.37		λ_3	.52			
λ_4	.56			λ_4		.53		.21
λ_5	.09		.37	λ_5			.17	.36
λ_6	.51	.41		λ_6	.65			
λ_7	.44	.44		λ_7	.61			
λ_8	.25		.25	λ_8			.42	
λ_9	.59	.45		λ_9	.74			
λ_{10}	.17		.51	λ_{10}			.30	.47
λ_{11}	.66			λ_{11}		.66		
λ_{12}	.54			λ_{12}		.53		
λ_{13}	.59	.46		λ_{13}	.75			
λ_{14}	.49	.40		λ_{14}	.63			
λ_{15}	.31		.26	λ_{15}			.52	
λ_{16}	.20		.21	λ_{16}			.38	
λ_{17}	.56			λ_{17}		.56		
λ_{18}	.10		.41	λ_{18}			.18	.42
λ_{19}	.17		.58	λ_{19}			.29	.56
λ_{20}	.21		.22	λ_{20}			.38	
				$r_{\text{DIF, DDF}}$	.78			
				$r_{\text{DDF, EOT}}$	.52			
				$r_{\text{DIF, EOT}}$	.39			
Model fit								
χ^2	6,236.8			χ^2	1,756.5			
df	155			*df*	162			
CFI	.953			CFI	.988			
RMSEA	.023			RMSEA	.012			
SRMR	.026			SRMR	.013			

	Model 7				
Factor(s)	DIF	DFF	EOT		
λ_1	.69			$r_{4,10}$	.10
λ_2		.75		$r_{5,10}$	.17
λ_3	.52			$r_{10,18}$	.19
λ_4		.53		$r_{4,18}$	.09
λ_5			.17	$r_{5,18}$	.15
λ_6	.65			$r_{10,19}$	.26
λ_7	.61			$r_{18,19}$	.24
λ_8			.42	$r_{4,19}$	.11
λ_9	.74			$r_{5,19}$	.20
λ_{10}			.30	$r_{4,5}$	.08
λ_{11}		.66			
λ_{12}		.53			
λ_{13}	.75				
λ_{14}	.63				
λ_{15}			.52		
λ_{16}			.38		
λ_{17}		.56			
λ_{18}			.18		
λ_{19}			.29		
λ_{20}			.38		
$r_{\text{DIF, DDF}}$	.78				
$r_{\text{DDF, EOT}}$	.52				
$r_{\text{DIF, EOT}}$	.39				
Model fit					
χ^2	1,753.9				
df	157				
CFI	.985				
RMSEA	.012				
SRMR	.013				

Note. N = 69,722. k = 88, DIF/DFF = difficulty identifying and describing feelings; DIF = difficulty identifying feelings; DDF = difficulty describing feelings; EOT = externally-oriented thinking; PT = pragmatic thinking; IOE = lack of (subjective significance or) importance of emotions. CFI = Comparative Fit Index; SRMR = Standardized Root Mean Square Residual; RMSEA = Root Mean Square Error of Approximation; In Model 3c the factor labels were retained, although the items load on other factors compared to the standard solution. Factors with # as superscript are associated with different interpretation across models although they have the same label.

Table OS 2

Factor Model Fit of the TAS-20 for The English Version

No	Model	χ^2	*df*	CFI	RMSEA	SRMR	AIC	BIC
1	Uni-dimensional model (Alex)	12,565.4	170	.803	.051 [b]	.097	12,225.4	10,821.5
2	Two-dimensional model (DIF/DDF – EOT)	6,760.9	169	.895	.037 [.036, .038]	.059	6,422.9	5,027.3
3a	Original three-dim. model (DIF – DDF – EOT)	3,378.0	167	.949	.026 [.025, .027]	.046	3,044.0	1,664.9
3b	Alternative three-dimensional model (DIF/DDF – PT – IOE)	6,674.8	167	.897	.037 [.036, .038]	.058	6,340.8	4,961.7
3c	Alternative three-dimensional model (DIF/DDF – EOT – IOE) [a]	8,689.4	167	.865	.042 [.042, .043]	.073	8,355.4	6,976.3
4	Four-dimensional model (DIF – DDF – PT – IOE)	3,291.4	164	.950	.026 [.025, .027]	.045	2,963.4	1,609.1
5	Bifactor model with three original nested factors	2,266.5	150	.966	.022 [.021, .023]	.025	1,966.5	727.8
6	Original three-dimensional model + nested method factor	312.5	162	.998	.006 [.005, .007]	.009	-11.6	-1,349.4
7	Original three-dimensional model + correlated residuals	311.8	157	.998	.006 [.005, .007]	.009	-2.3	-1,298.8

Note. N = 28,514. k = 34, DIF/DFF = difficulty identifying and describing feelings; DIF = difficulty identifying feelings; DDF = difficulty describing feelings; EOT = externally-oriented thinking; PT = pragmatic thinking; IOE = lack of (subjective significance or) importance of emotions. CFI = Comparative Fit Index; SRMR = Standardized Root Mean Square Residual; RMSEA = Root Mean Square Error of Approximation; AIC = Akaike Information Criterion; BIC = Bayesian Information Criterion.

[a] In this solution, the factor labels were retained, although the items load on other factors compared to the standard solution.

[b] Confidence interval could not be computed.

Table OS 3

Parameter Estimates of the TAS-20 Across Psychiatric Status

	Non-clinical				Clinical		
Factor(s)	DIF	DDF	EOT		DIF	DDF	EOT
λ_1	.71			λ_1	.69		
λ_2		.74		λ_2		.73	
λ_3	.53			λ_3	.51		
λ_4		.58		λ_4		.47	
λ_5			.30	λ_5			.29
λ_6	.70			λ_6	.69		
λ_7	.62			λ_7	.58		
λ_8			.36	λ_8			.37
λ_9	.76			λ_9	.74		
λ_{10}			.58	λ_{10}			.36
λ_{11}		.68		λ_{11}		.59	
λ_{12}		.58		λ_{12}		.50	
λ_{13}	.77			λ_{13}	.73		
λ_{14}	.66			λ_{14}	.67		
λ_{15}			.44	λ_{15}			.45
λ_{16}			.30	λ_{16}			.35
λ_{17}		.59		λ_{17}		.51	
λ_{18}			.39	λ_{18}			.43
λ_{19}			.43	λ_{19}			.49
λ_{20}			.31	λ_{20}			.33
$r_{\text{DIF, DDF}}$	.81			$r_{\text{DIF, DDF}}$	.78		
$r_{\text{DDF, EOT}}$	.51			$r_{\text{DDF, EOT}}$	.54		
$r_{\text{DIF, EOT}}$	.39			$r_{\text{DIF, EOT}}$	.36		
Model fit							
N	27,453	CFI	.948	N	1,061	CFI	1.00
χ^2	3,368.3	RMSEA	.026	χ^2	88.0	RMSEA	.000
df	167	SRMR	.048	df	167	SRMR	.026

Note. k = 34, DIF/DFF = difficulty identifying and describing feelings; DIF = difficulty identifying feelings; DDF = difficulty describing feelings; CFI = Comparative Fit Index; SRMR = Standardized Root Mean Square Residual; RMSEA = Root Mean Square Error of Approximation.

Figure OS 1

Correlation Matrix of All Correlation Matrices

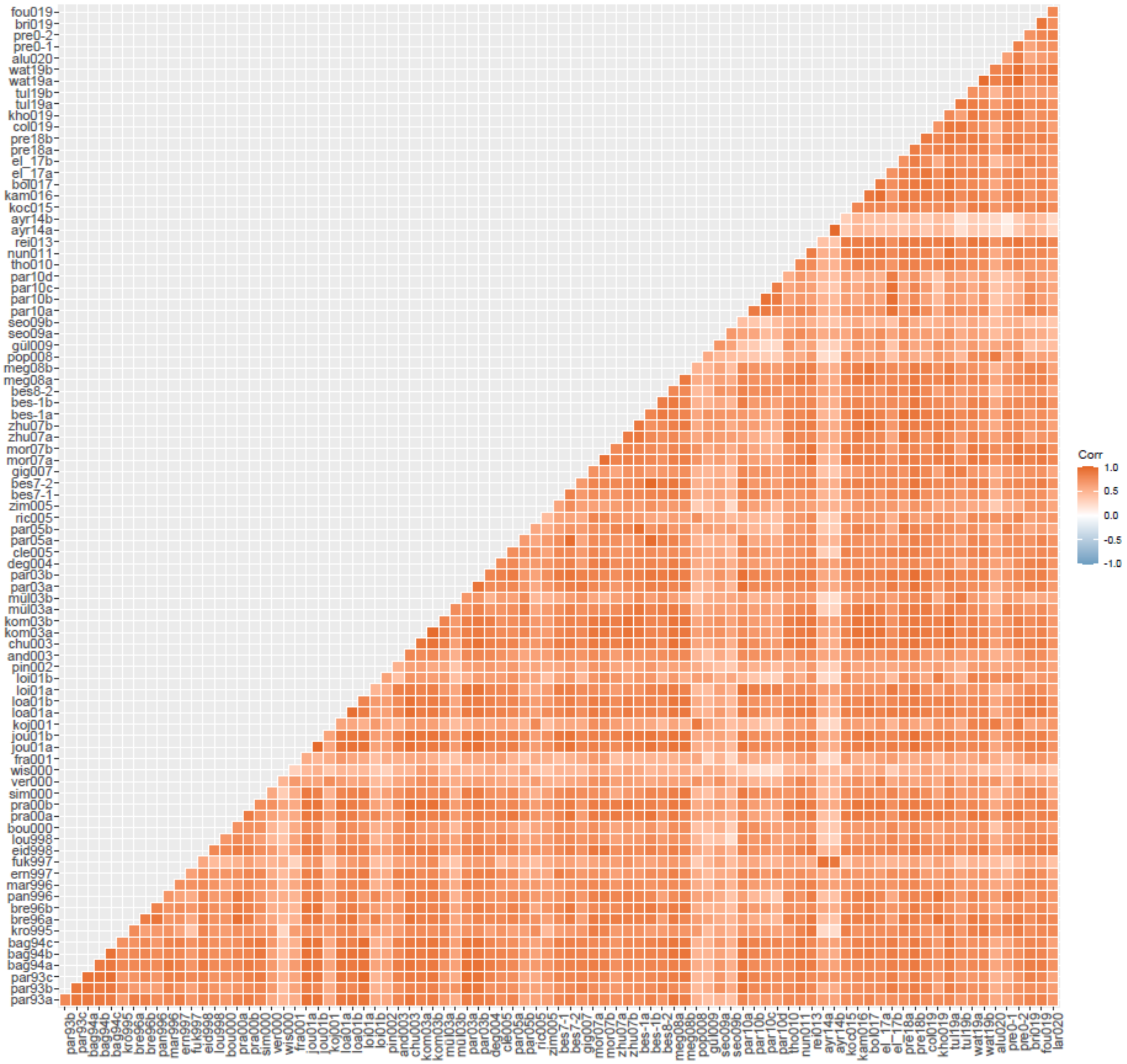

Supplement: sj-pdf-1-asm-10.1177_10731911211033894 – Supplemental material for The Structure of the Toronto Alexithymia Scale (TAS-20): A Meta-Analytic Confirmatory Factor Analysis [file sj-pdf-1-asm-10.1177_10731911211033894.pdf]
